# ASIC1α up-regulates MMP-2/9 expression to enhance mobility and proliferation of liver cancer cells via the PI3K/AKT/mTOR pathway

**DOI:** 10.1186/s12885-022-09874-w

**Published:** 2022-07-16

**Authors:** Yinci Zhang, Jiaojiao Liang, Niandie Cao, Jiafeng Gao, Yinghai Xie, Shuping Zhou, Xiaolong Tang

**Affiliations:** 1 Medcial School, Anhui University of Science & Technology, Huainan, 232001 China; 2 Institute of Environment-Friendly Materials and Occupational Health of Anhui, University of Science and Technology, Wuhu, 241003 China; 3 First Affiliated Hospital, Anhui University of Science & Technology, Huainan, 232001 China

**Keywords:** Liver cancer, Acid-sensitive ion channel 1α, PI3K/AKT/mTOR, MMP-2/9, HepG2 cells, SK-Hep1cells

## Abstract

A major challenge in the treatment of liver cancer is that a large proportion of patients fail to achieve long-term disease control, with death from liver cancer cell migration and invasion. Acid-sensitive ion channel 1α (ASIC1α) is involved in the migration, invasion, and proliferation of liver cancer cells. Therefore, we explored the mechanism of ASIC1α-mediated liver cancer cell migration and invasion. We determined the levels of ASIC1α by western blotting and immunofluorescence in HepG2 and SK-Hep1 cells cultured in various acidic conditions. In addition, wound healing assay, transwell invasion assay, and MTT assay were conducted to assess the migration, invasion, and proliferation abilities of liver cancer cells. Western blotting was conducted to determine the levels of MMP2, MMP9, ASIC1α, p-PI3Kp85, t-PI3Kp85, p-AKT(Ser473), t-AKT, p-mTOR (Ser2448), t-mTOR. We first found that the levels of ASIC1α in the HepG2 and SK-Hep1 cells in acidic conditions (pH 6.5) were significantly increased. Inhibition and knockdown of ASIC1α down-regulated MMP-2/9 expression and inhibited the migration, invasion, and proliferation of HepG2 and SK-Hep1 cells; overexpression of ASIC1α had the opposite effect. We further demonstrated that ASIC1α up-regulates MMP-2/9 via activation of the PI3K/AKT/mTOR pathway, thereby promoting migration, invasion, and proliferation of liver cancer cells. Overexpression of MMP-2/9 and activation of AKT reversed these effects on liver cancer cells caused by inhibition of ASIC1α. We conclude that ASIC1α can regulate migration, invasion, and proliferation of liver cancer cells through the MMP-2/9/PI3K/AKT/mTOR pathway. These observations may provide a new reference for liver cancer chemotherapy.

**Supplementary Information:**

The online version contains supplementary material available at 10.1186/s12885-022-09874-w.

## Introduction

Liver cancer is the most common lethal malignancy in the world [1]. Malignant progression and metastasis are serious problems in the treatment and prognosis of patients with liver cancer. Migration and invasion are major features of tumour cells, and they contribute much to the malignant progression and metastasis of tumours. Therefore, clarification of the mechanism of liver cancer migration and invasion may help toward developing treatment strategies that could improve the prognosis of liver cancer patients.

Acid-sensing ion channels (ASICs) are cation-permeable protein complexes that belong to the epithelial-channel mutagenic protein ion channel superfamily. ASICs play an important role in sensing the pH of body fluids and regulating physiological functions such as pain and sour taste. ASIC1α is involved in pathological behaviours and regulation of biological functions that are identified with ASIC subunits 1α, 1b, 2α, 2b, 3, and 4 [2–6]. The high glucose metabolic rate in tumour patients, combined with poor perfusion, results in an acidic tumour microenvironment with a pH of about 6.5 [7, 8]. Thus, the acid-sensitive ion-channel molecule, ASIC1α, may be associated with cancer and is involved in the proliferation and migration of tumour cells [9–12]. ASIC1α is upregulated in liver cancer cells and can promote the migration and invasion of liver cancer cells [9, 13]. However, the molecular mechanisms and pathways involved in the migration and invasion of liver cancer cells by ASIC1α have not been defined.

Matrix metalloproteinases (MMPs) maintain the homeostasis of extracellular matrices and play a key role in tissue remodelling, organogenesis, inflammation, and tumour progression [14–16]. MMP2/9 is an important regulator of the degradation of the tumour basement membrane and extracellular matrix, thereby promoting cell migration and invasion [17–19]. High levels of MMP2/9 are associated with increased metastasis and poor prognosis in liver cancer patients [20–22]. However, the relationship between ASIC1α and MMP-2 and MMP-9 in liver cancer cells has not been elucidated.

Abnormal activation of the PI3K/AKT/mTOR pathway has a key role in biological behaviours, such as proliferation, migration, invasion, and apoptosis of tumour cells [23–26]. Down-regulation of the PI3K/AKT/mTOR/MMP-2/9 pathway can inhibit migration and invasion of tumour cells [20, 21]. ASIC1α also has been involved in the proliferation and migration of tumour cells [9–12]. Whether ASIC1α can inhibit the migration, invasion, and proliferation of liver cancer by inhibiting MMP-2 and MMP-9 via the PI3K/AKT/mTOR pathway is unknown.

In this study, we identified the function of ASIC1α, explored the role of ASIC1α in the migration, invasion, and proliferation of HepG2 and SK-Hep1 cells, and elaborated on a molecular mechanism involved. This information may lead to a new way to control the progression of malignant liver cancer and to treat patients with malignant liver cancer to improve their prognosis.

## Materials and methods

### Preparation of acidic (pH 6.5) medium

Concentrated hydrochloric acid was slowly added to 20 mL of normal culture (pH 7.4) RPMI 1640 medium, and the pH was determined with a pH meter. When the pH reached 6.5, the added amount of concentrated hydrochloric acid was recorded. Then, concentrated hydrochloric acid was added to the RPMI 1640 medium according to the volume ratio of the medium to the concentrated hydrochloric acid to achieve a medium with pH 6.5.

### Cell source and culture

The human liver cell line L-02 was received from the Chinese Academy of Sciences (Shanghai, China); the human liver cancer cell line SK-Hep1 was obtained from Cellcook Biotech Co., Ltd. (Guangzhou, China); and the HepG2 cell line was acquired from Mingjin Biotech Co., Ltd. (Shanghai, China). The cell lines were cultured in RPMI 1640 medium supplemented with 10% FBS at 37 ℃ with 5% CO_2_. The HepG2 and SK-Hep1 cells were cultured in acidic (pH 6.5) medium for 4 h to activate ASIC1a.

### Reagents, antibodies, and chemicals

A potent and selective ASIC1α blocker, Psalmotoxin 1 (PcTx1) (#B5796), and a type IV collagen-specific MMP-2 and MMP-9 inhibitor, cis-ACCP (#C4501), were purchased from APExBIO Technology LLC (Houston, TX, USA). LY294002 (a broad-spectrum PI3K inhibitor) (#S1105), MK-2206 (a AKT inhibitor) (#S1078), Rapamycin (a specific inhibitor of mTOR) (#S1039) and NVP-BEZ235 (a dual PI3K/mTOR inhibitor) (#S1009) were obtained from Selleck Chemicals (Houston, TX, USA). SC79 (a brain-permeable AKT phosphorylation activator) (#HY-18749) was obtained from MedChem Express (New Jersey, USA). Primary antibodies for p-PI3Kp85 (#4228), t-PI3Kp85 (#4292), p-AKT(Ser473) (#4060), t-AKT (#4691), p-mTOR(Ser2448) (#2971) and t-mTOR (#2972) were purchased from Cell Signaling Technology (Danvers, MA, USA). Antibodies to MMP-2 (#ab92536) and MMP-9 (#ab283575) were obtained from Abcam Biological Technology (Cambridge, UK). Antibody against ASIC1a (#ASC-014) was purchased from Alomone Labs Company (Jerusalem, Israel). Antibody to β-actin (#AF5003) was received from Biosharp Life Science (Hefei, China). The BCA-200 protein assay kit (#23,227) was obtained from Thermo Scientific (Rockford, IL, USA). Phosphatase inhibitor tablets (#P1045) were obtained from Beyotime Biotechnology (Shanghai, China). ASIC1α-specific shRNA lentiviral particles, ASIC1α gene-lentiviral particles, MMP-2 gene-lentiviral particles, and MMP-9 gene-lentiviral particles were obtained from Genechem Co., Ltd (Shanghai, China).

### Indirect immunofluorescence analysis

HepG2, SK-Hep1, and L-02 cells were washed three times with PBS, fixed with 4% paraformaldehyde for 15 min, permeated with 0.5% Triton-100 for 10 min, and blocked with 5% BSA for 1 h at room temperature. Anti-ASIC1α (1:200) was incubated at 4 °C overnight, and Alexa Fluor 488-conjugated goat anti-rabbit IgG (1:1000) was incubated in the dark for 1 h at room temperature. Finally, the cells were stained with DAPI (4',6 diamidino-2-phenylindole, dihydrochloride) for 15 min and imaged with an inverted fluorescence microscope.

### Silencing of ASIC1α

The ASIC1a-specific shRNA lentiviral particles were transfected into cells according to the manufacturer’s protocol. The shRNA for gene silencing are

ASIC1a shRNA: CCGGCTATGGAAAGTGCTACACGTTC

TCGAGAACGTGTAGCACTTTCCATAGTTTTT.

### Overexpression of MMP-2, MMP-9 and ASIC1α

The MMP-2, MMP-9, and ASIC1a lentiviral particles was transfected into cells as according to the manufacturer’s protocol. The primers for MMP-2, MMP-9 and ASIC1a overexpression are

5′-GATACCCCTTTGACGGTAAGGA-3′ (forward)

and 5′-CCTTCTCCCAAGGTCCATAGC-3′ (reverse);

5′-GTGCTGGGCTGCTTTGCTG-3′ (forward) and

5'-GTCGCCCTCAAAGGTTTGGAAT-3′ (reverse);

5′-GAGGATCCCCGGGTACCGGTCGCCACCAT

GGAACTGAAGGCCGAGGAGGAG-3′ (forward)

5′-TCCTTGTAGTCCATACCGCAGGTAAAGTCC

TCGAACGTG-3′ (reverse), respectively.

### Wound healing assay

HepG2, SK-Hep1, and L-02 cells were inoculated into six-well plates with 90% confluence the next day. The cells were scraped with a 20 µL plastic tip. After the cells were washed with PBS, photographs were taken at 0 h and 48 h. Scratch-healing ability was expressed as migration area relative to 0 h.

### Invasion assay

BD Matrigel (Solarbio, Shanghai) was held at 4 °C to become liquid and added to the serum-free medium at a ratio of 1: 5 and mixed (at 4 °C, preferably on an ice bath). One hundred microliters of the mixed solution were added to the upper chamber and placed in a 37 °C incubator for 5 h. The cells were digested, counted, and prepared for cell suspension. The experiment thereafter proceeded in accordance with our described method [27].

### MTT assay

Cells were inoculated into 96-well plates at a density of 1 × 10^5^ cells/well overnight, and 10 µL MTT solution (5 mg/mL, 0.5% MTT) were added and cultured for 4 h. Dimethylsulfoxide (DMSO; 150 µL) was added and measured at OD 490 nm. Blank wells (medium, MTT, DMSO) and control wells (cells, drug dissolution medium of the same concentration, culture solution, MTT, DMSO) were set. Cell viability was calculated as (OD in test well-OD in blank well) / (OD in control well-OD in blank well) × 100%.

### Western blot

Cultured cells were lysed in microcentrifuge tubes containing a mixture of protease and phosphatase inhibitors. The protein concentration was determined with a BCA-200 Protein Assay Kit. Various concentrations of SDS-PAGE were used to separate the protein samples, which were electrotransferred onto PVDF membranes. After sealing for 1 h at room temperature with 5% skim milk dissolved in TBST, the membranes were reacted overnight at 4 °C, followed by the incubation of secondary antibodies at room temperature for one hour. The density of the target band was quantified with an ImageJ digital imaging system (http://imagej.nih.gov/ij/). Due to insufficient experimental funds, in the western blot assay, we cut the PVDF membrane into a membrane small enough to incubate the antibody according to the molecular weight of the incubated antibody and the protein molecular weight marker. In addition, protein samples of related research topics that were conducted simultaneously were added to the same gel for protein electrophoresis.

### Statistical analysis

All experiments were performed at least in triplicate and measured in three independent experiments. The data were expressed as mean ± SD. Comparison between means of two groups was performed with Student's t-test, and comparison among three or more groups was performed with analysis of one-way ANOVA. *P* < 0.05 was considered statistically significant. GraphPad Prism 5 was used for all analyses.

## Results

### High expression of ASIC1α in HepG2 and SK-Hep1 cells cultured at pH 6.5

**Fig. 1:**
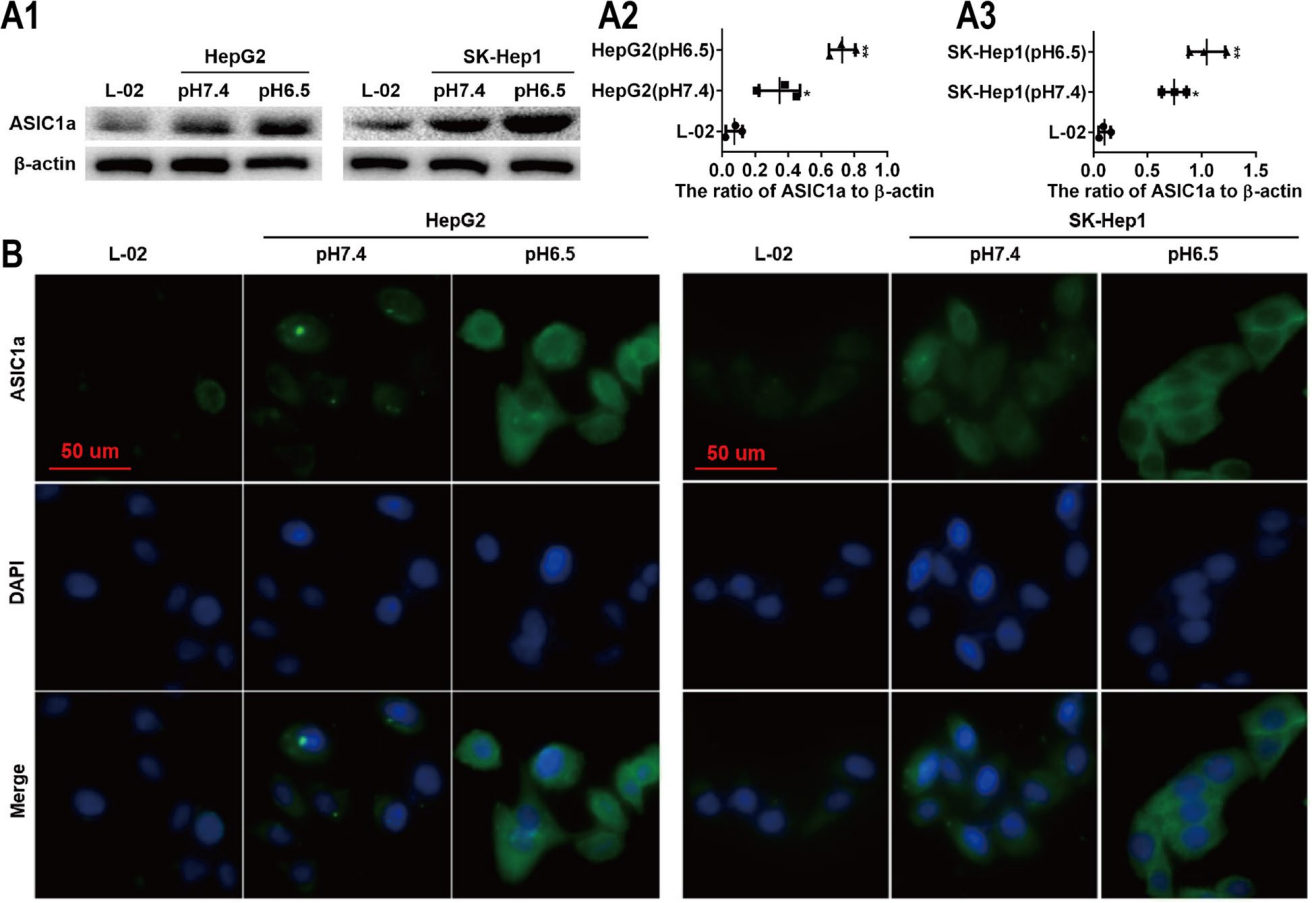
High expression of ASIC1α in HepG2 and SK-Hep1 cells which cultured in pH6.5. **A1** Expression levels of ASIC1α in human liver cancer cell lines and a normal L-02 hepatic cell line were detected by western blotting. **A2** and **A3** Histogram showing the semiquantitative analyses of the gels from western blotting. **B** Expression levels and the cell distribution of ASIC1α in human liver cancer cell lines and a normal L-02 hepatic cell line were measured by immunofluorescence. Representative images were taken at × 400 magnification. Data were expressed as the mean ± SD, *n *= 3. *P < 0.05 and **P < 0.01 all versus L-02 group

We first measured the expression levels of ASIC1α in the L-02, HepG2, and SK-Hep1 cells cultured in physiologic (pH 7.4) or pH 6.5 medium. As shown in Figs. 1A1, A2, and A3, the ASIC1α protein was highly expressed in the liver cancer cells, especially in those cultured in pH 6.5 medium, a finding consistent with that in a previous study [7, 8]. We also used immunofluorescence assay to study the intracellular distribution of ASIC1α in liver cancer cells. As displayed in Fig. 1B, ASIC1α was mainly present in the plasma membrane. In addition, consistent with the results of western blotting, ASIC1α staining was more intense in HepG2 and SK-Hep1 cells cultured at pH 6.5 and pH 7.4 than in the L-02 cells, especially in those cultured at pH 6.5. These data showed that up-regulation of ASIC1α was positively correlated with the progression of liver cancer in an acidic microenvironment.

**Fig. 2:**
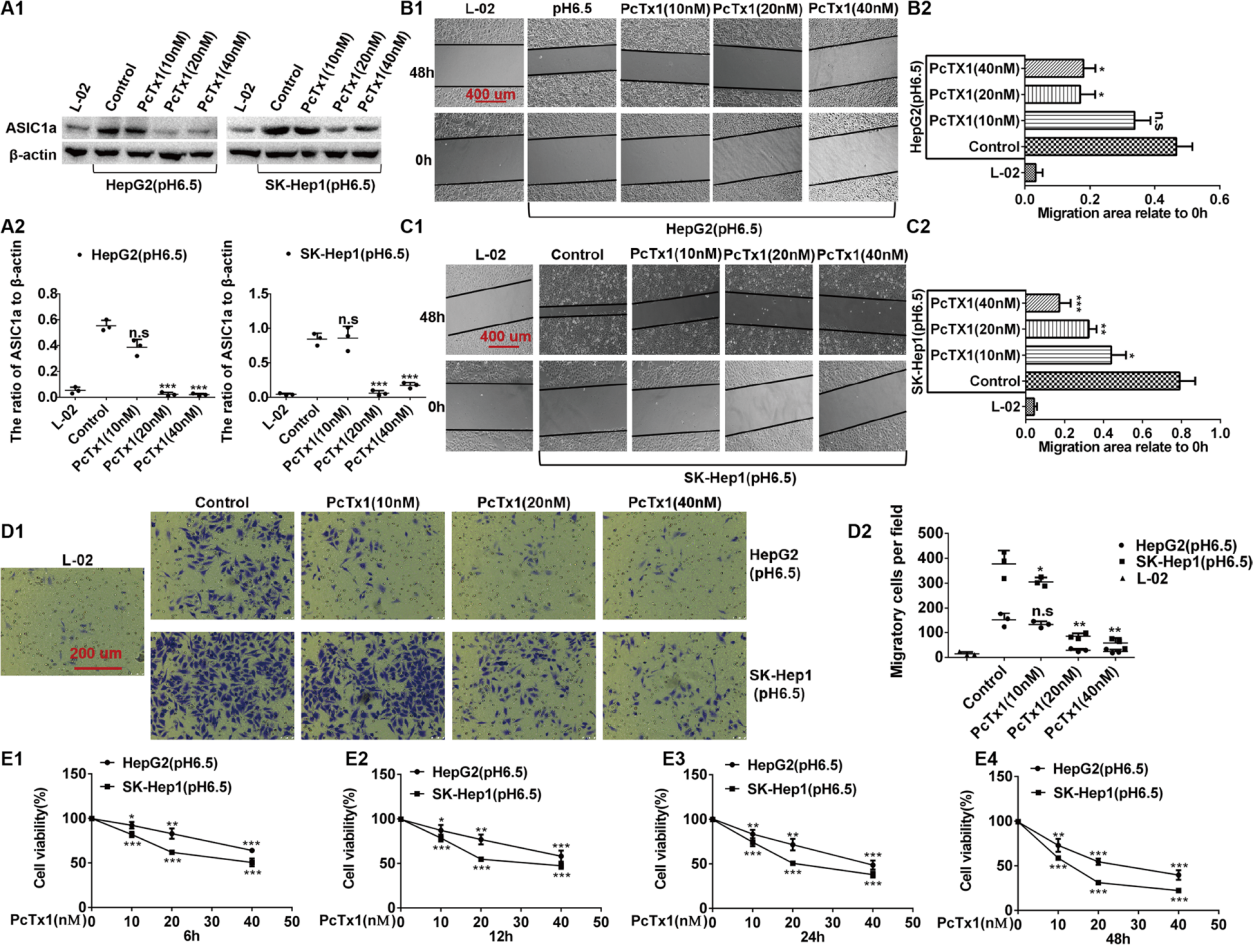
Inhibiting the activity of ASIC1α reduces the mobility and proliferation of HepG2 and SK-Hep1 Cells. **A1** and **A2** Expression levels of ASIC1α in different group cells were detected by western blotting. The concentrations of PcTx1 were 10, 20, 40 nM at 24 h. **B1**, **B2**, **C1** and **C2** Migration abilities of different group cells were examined by wound healing assays. Representative images were taken at × 100 magnification. The cells were treated with PcTx1 for 6 h before performing wound healing assays. **D1** and **D2** Invasion abilities of different group cells were determined by transwell invasion assays. Representative images were taken at × 200 magnification. Invasion assays were conducted at 24 h. The cells were treated with PcTx1 for 6 h before performing transwell invasion assays. **E1**, **E2**, **E3** and **E4** proliferation abilities of different group cells were determined by MTT assay. Data were expressed as the mean ± SD, *n* = 3. n.s: no significance, *P < 0.05, **P < 0.01 and ***P < 0.001 all versus pH6.5 group or PcTx1(0 nM) group

### Inhibiting the activity of ASIC1α reduces the mobility and proliferation of HepG2 and SK-Hep1 Cells

The ability of PcTx1 (a potent and selective ASIC1α blocker) to inhibit ASIC1α in the HepG2 and SK-Hep1 liver cancer cells was measured with western blotting. When the concentration of PcTx1 reached 20 nM, ASIC1α was inhibited in pH 6.5 medium (Figs. 2A1 and A2). The data in Figs. 2B1, B2, 2C1, and 2C2 reveal that 20 nM PcTx1 inhibited the migration of HepG2 cells (*P* < 0.05), whereas 10 nM PcTx1inhibited the migration of SK-Hep1 cells (*P* < 0.01). Transwell invasion assays were then used to analyze invasion of the cells. As displayed in Figs. 2D1 and D2, 10 nM PcTx1 did not inhibit invasion of cells in SK-Hep1 cells (n.s), but when the concentration of PcTx1 reached 20 nM, invasion was significantly inhibited in HepG2 and SK-Hep1 cells (*P* < 0.01). (Note: the cells were treated with PcTx1 for 6 h before performing wound healing assays and transwell invasion assays.) Lastly, MTT assay was used to analyze proliferation of various groups of cells. As illustrated in Figs. 2E1, E2, E3, and E4, 10 nM PcTx1 did not inhibit cell proliferation, (n.s), but at 20 nM PcTx1, proliferation of SK-Hep1 cells was significantly inhibited, and at 40 nM PcTx1, the proliferation of HepG2 cells was significantly inhibited. These results documented that liver cancer cells with a higher ASIC1α expression level had enhanced migration, invasion, and proliferation, whereas inhibiting the expression of ASIC1α reduced the migration, invasion, and proliferation.

### Silencing the ASIC1α gene results in decreased mobility and proliferation of HepG2 and SK-Hep1 cells

**Fig. 3:**
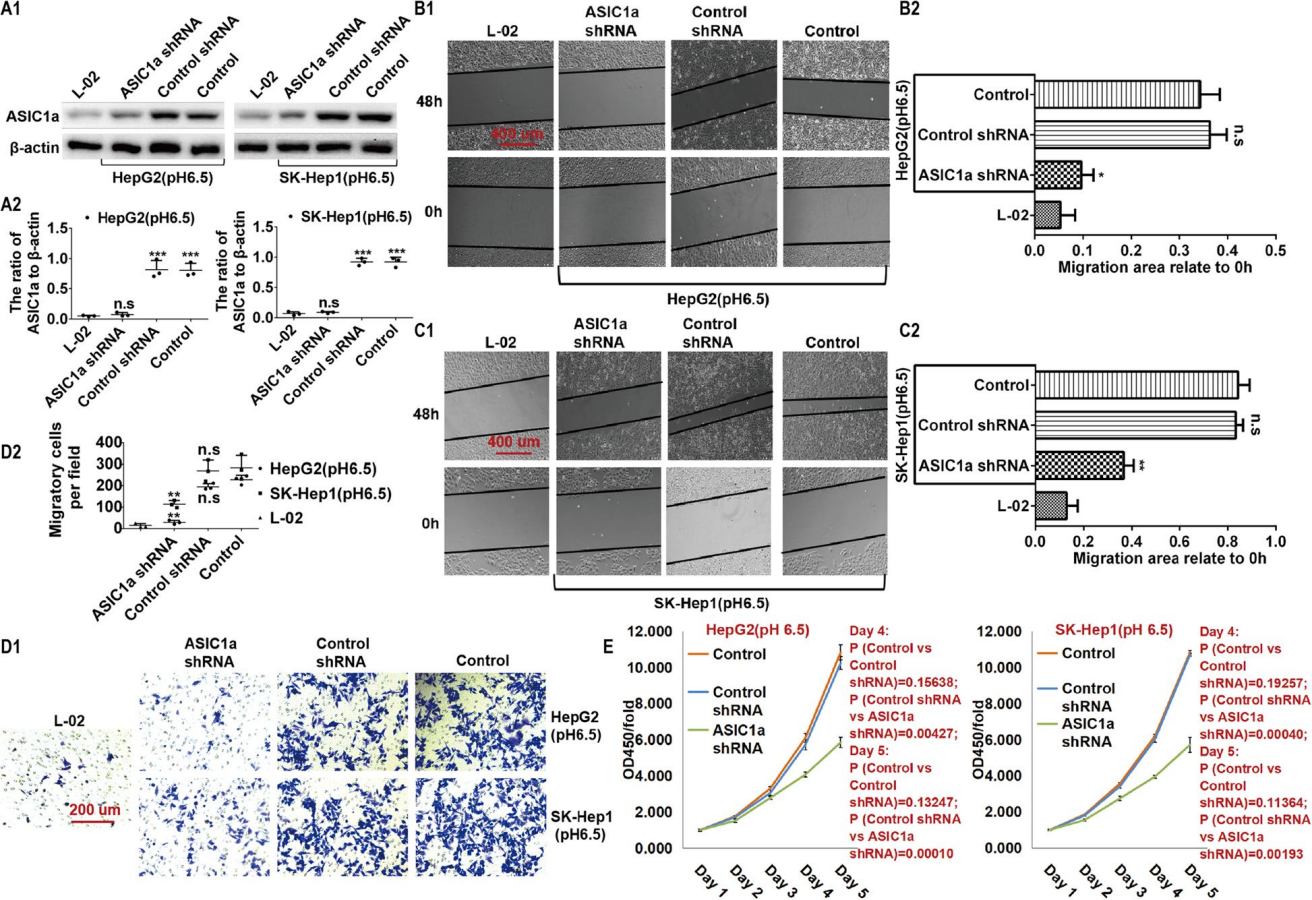
Silencing of the ASIC1α gene inhibits the mobility and proliferation of HepG2 and SK-Hep1 cells. **A1** and **A2** Expression levels of ASIC1α in different group cells were detected by western blotting. **B1**, **B2**, **C1** and **C2** Migration abilities of different group cells were examined by wound healing assays. Representative images were taken at × 100 magnification. **D1** and **D2** Invasion abilities of different group cells were determined by transwell invasion assays. Representative images were taken at × 200 magnification. Invasion assays were conducted at 24 h. **E** proliferation abilities of different group cells were determined by MTT assay. Data were expressed as the mean ± SD, *n* = 3. n.s: no significance, *P < 0.05, **P < 0.01 and ***P < 0.001 all versus pH6.5 group or L-02 group

Since the expression of ASIC1α was significantly upregulated in liver cancer cells cultured in pH 6.5 medium, we silenced the ASIC1α gene by shRNA to study the effect of the ASIC1α protein on migration, invasion, and proliferation of the HepG2 and SK-Hep1 cells. The silencing effect was detected by western blotting. As shown in Figs. 3A1 and A2, ASIC1α was silenced by ASIC1α shRNA transfection. The migration of HepG2 cells was then evaluated with the wound healing assay. Cell migration was significantly decreased (*P* < 0.05) in the HepG2 (pH 6.5)-shASIC1α cells compared with migration in the HepG2 (pH 6.5) cells (Figs. 3B1 and B2). Similar results were obtained when SK-Hep1 (pH 6.5) cells were treated with ASIC1α shRNA (Figs. 3C1 and C2). We next applied transwell assays to examine the invasion of HepG2 and SK-Hep1 cells after silencing ASIC1α. As displayed in Figs. 3D1 and D2, cell invasion was significantly decreased (*P* < 0.01) in the HepG2 (pH 6.5)-shASIC1α cells compared with invasion in the HepG2 (pH 6.5) cells. Similar results were obtained with SK-Hep1 cells. Lastly, we used the MTT assay to test the proliferation of HepG2 and SK-Hep1 cells after treating them at 5 time points (1, 2, 3, 4, and 5 days after cell inoculation). As shown in Fig. 3E, cell proliferation was decreased after silencing ASIC1α, which indicated that upregulated expression level of ASIC1α resulted in increase proliferation of the cells.

### Overexpression of the ASIC1α gene resulted in increased mobility and proliferation of HepG2 and SK-Hep1 cells

**Fig. 4:**
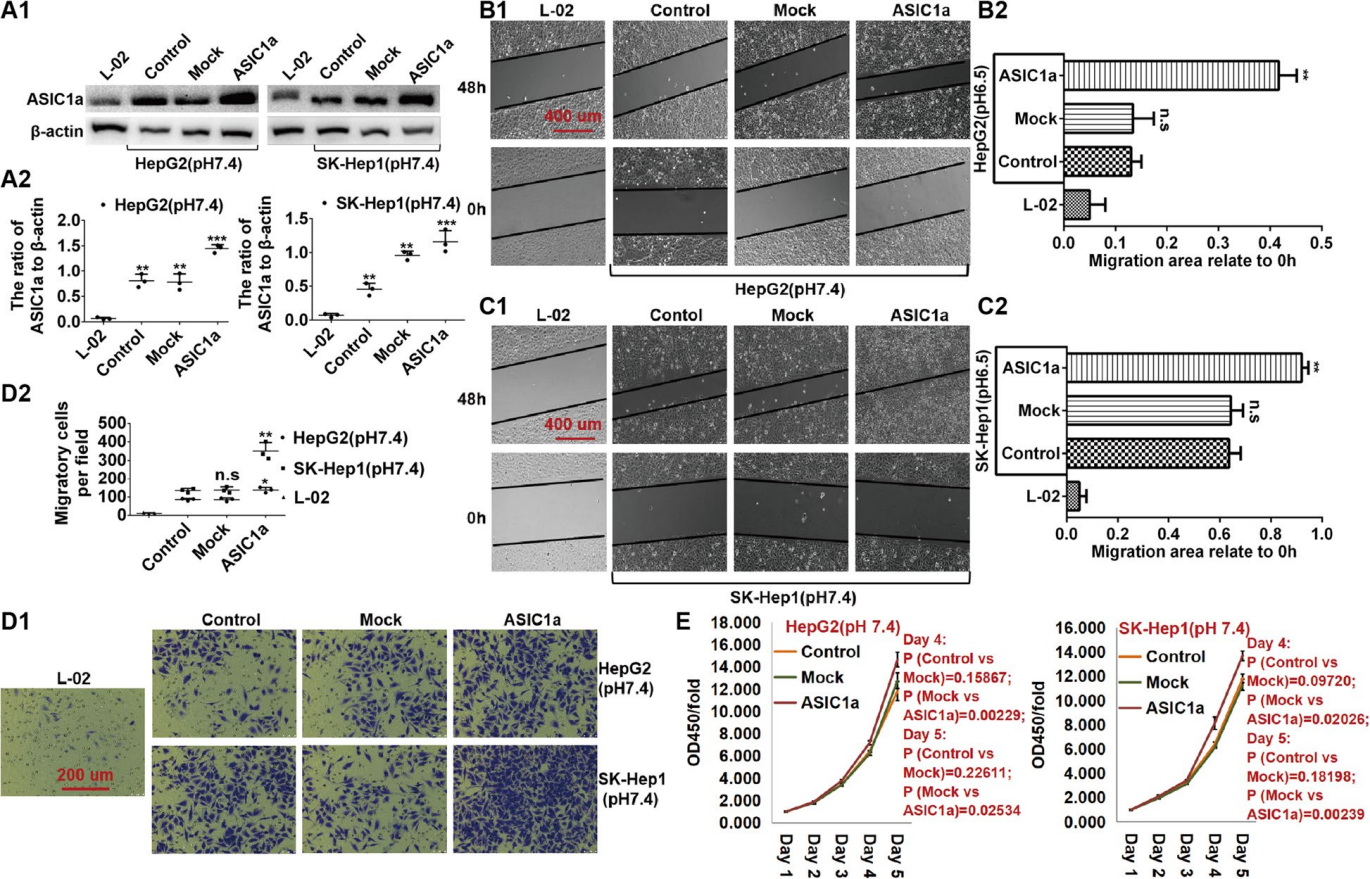
Overexpression of the ASIC1α gene enhances the mobility and proliferation of HepG2 and SK-Hep1 cells. **A1** and **A2** Expression levels of ASIC1α in different group cells were detected by western blotting. **B1**, **B2**, **C1** and **C2** Migration abilities of different group cells were examined by wound healing assays. Representative images were taken at × 100 magnification. **D1** and **D2** Invasion abilities of different group cells were determined by transwell invasion assays. Representative images were taken at × 200 magnification. Invasion assays were conducted at 24 h. **E** proliferation abilities of different group cells were determined by MTT assay. Data were expressed as the mean ± SD, *n* = 3. n.s: no significance, *P < 0.05, **P < 0.01 and ***P < 0.001 all versus pH6.5 group or L-02 group

ASIC1α was overexpressd by ASIC1α transfectants, as illustrated in Figs. 4A1 and A2. We then showed that the migration ability of HepG2 cells (*P* < 0.01) (Figs. 4B1 and B2) and SK-Hep1 cells (*P* < 0.01) (Figs. 4C1 and C2) was increased by overexpression of ASIC1α. We also examined the effects of overexpression of ASIC1α on the invasion ability of HepG2 cells and SK-Hep1 cells at pH 7.4 with transwell invasion assays. Compared with HepG2 cells, the invasion ability of HepG2-ASIC1α cells was significantly increased (*P* < 0.05) (Figs. 4D1 and D2), and, compared with the invasion ability of SK-Hep1 cells, invasion of SK-Hep1-ASIC1α cells was significantly increased (*P* < 0.01). Thus, ASIC1α overexpression resulted in increased migration and invasion of both liver cancer cell lines, HepG2 and SK-Hep1. Finally, we tested the proliferation ability of the liver cancer cells with MTT assay at 5 time points (1, 2, 3, 4, and 5 days after cell inoculation) and found that overexpression of ASIC1α enhanced proliferation of the cells (Fig. 4E).

### Inhibiting the activity of ASIC1α reduces the mobility and proliferation of HepG2 and SK-Hep1 cells by down-regulating the expression of MMP-2 and MMP-9

**Fig. 5:**
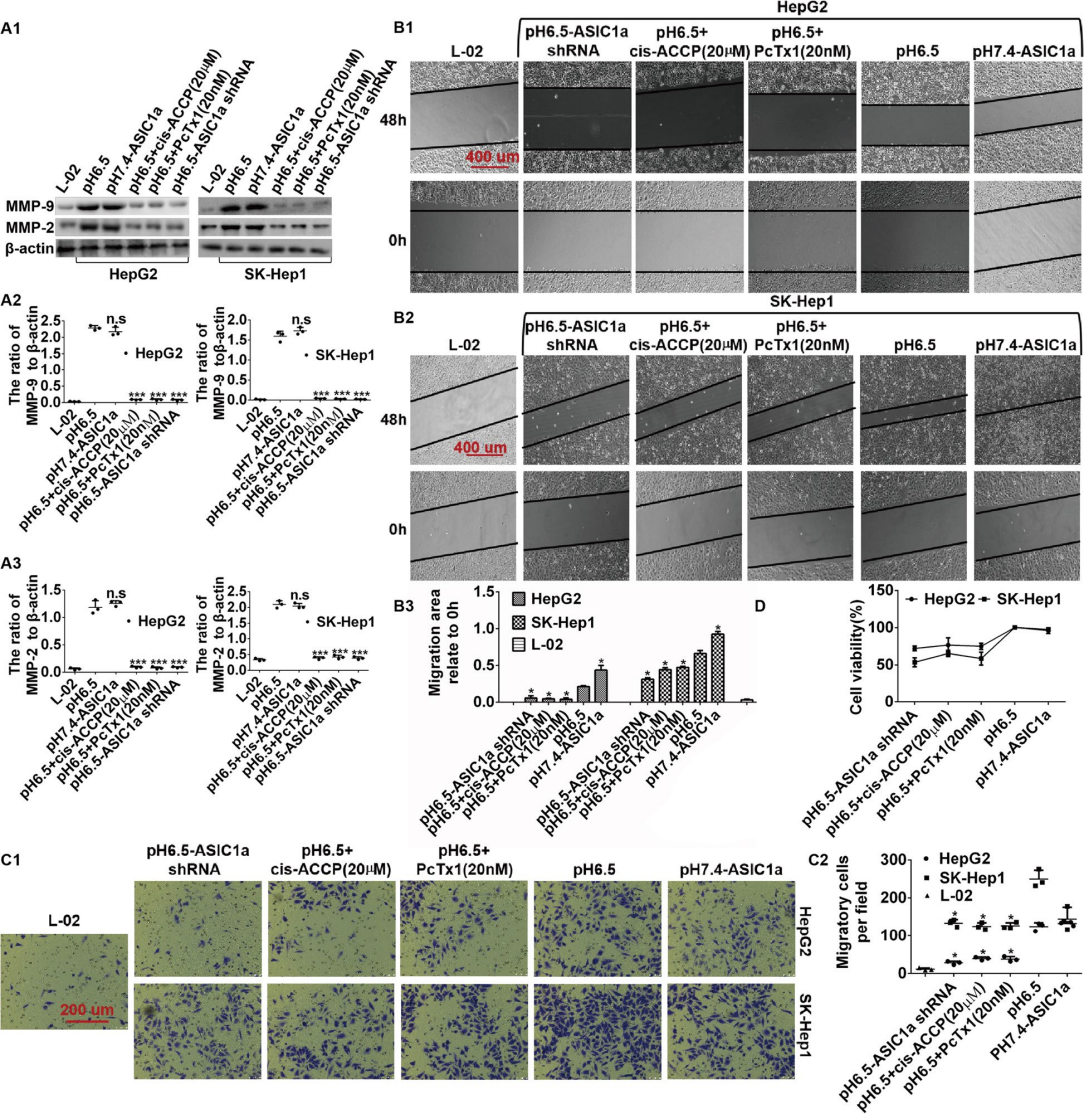
Inhibiting the activity of ASIC1α reduces the mobility and proliferation of HepG2 and SK-Hep1 Cells by down-regulating the expression of MMP-2 and MMP-9. **A1**, **A2** and **A3** Expression levels of MMP-2 and MMP-9 in different group cells were detected by western blotting. The concentration of PcTx1 was 20 nM, and the concentration of cis-ACCP was 20 µM at 24 h. **B1**, **B2** and **B3** Migration abilities of different group cells were examined by wound healing assays. Representative images were taken at × 100 magnification. The cells were treated with PcTx1 or cis-ACCP for 6 h before performing wound healing assays. **C1** and **C2** Invasion abilities of different group cells were determined by transwell invasion assays. Invasion assays were conducted at 24 h. The cells were treated with PcTx1 or cis-ACCP for 6 h before performing transwell invasion assays. Representative images were taken at × 200 magnification. **D** proliferation abilities of different group cells were determined by MTT assay. MTT assays were conducted at 24 h. Data were expressed as the mean ± SD, *n* = 3. n.s: no significance, *P < 0.05 and ***P < 0.001 all versus pH6.5 group

From the above experimental results, we confirmed that ASIC1α mediates migration, invasion, and proliferation of liver cancer cells in pH 6.5, which is consistent with the results of previous studies [7, 8]. Since MMP-2/9 plays an important role in the migration and invasion of liver cancer cells and is highly expressed in liver cancer cells, we explored the relationship between ASIC1α and MMP-2/9. First, we measured the level of MMP-2/9 in each cell type (Figs. 5A1, A2 and A3). Compared with HepG2 (pH 6.5) cells, the expression levels of MMP-9 (*P* < 0.001) and MMP-2 (*P* < 0.001) in HepG2 (pH 6.5) + cis-ACCP (a type IV collagen-specific MMP-2 and MMP-9 inhibitor), HepG2 (pH 6.5) + PcTx1 (20 nM) group, and HepG2 (pH6.5)-ASIC1α shRNA group were significantly decreased, while the expression levels of MMP-9 and MMP-2 in the HepG2 (pH 7.4)-ASIC1α cells were not significantly different. Similar results were obtained with SK-Hep1 cells. These data indicated that ASIC1α could regulate the expression of MMP-2/9. Next, we examined the migration ability of liver cancer cells. As shown in Figs. 5B1, B2, and B3, compared with the HepG2 (pH 6.5) group, the migration area relative to 0 h of HepG2 (pH6.5) + cis-ACCP group, HepG2 (pH 6.5) + PcTx1 (20 nM) group, HepG2 (pH 6.5)-ASIC1α shRNA group were significantly decreased (*P* < 0.05). Similar results were obtained with SK-Hep1 (pH 6.5) cells. We also examined the invasion abilities of HCC cells, as shown in Figs. 5C1 and C2, with transwell invasion assay. Compared with HepG2 (pH 6.5) cells, the number of migrated cells per field was decreased (*P* < 0.05) in HepG2 (pH 6.5) + cis-ACCP cells, HepG2 (pH 6.5) + PcTx1(20 nM) cells, and HepG2 (pH6.5)-ASIC1α shRNA cells. Similar results were obtained with SK-Hep1 (pH 6.5) cells. (Note: The cells were treated with PcTx1 or cis-ACCP for 6 h before wound healing assays and transwell invasion assays were performed.) We then measured the proliferation ability of the various cells with MTT assay. ASIC1α treatment resulted in enhanced proliferation ability of liver cancer cells (Fig. 5D). Thus, the above results documented that inhibiting the activity of ASIC1α reduces the mobility and proliferation ability of HepG2 and SK-Hep1 cells by reducing the amount of MMP2/9.

**Fig. 6:**
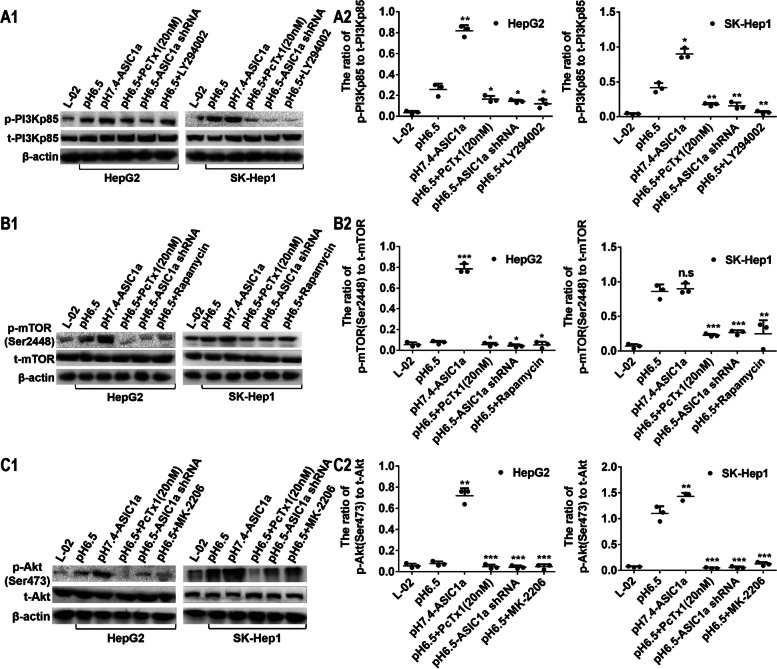
ASIC1a can regulate aberrant activation of the PI3K/AKT/MTOR pathway. **A1**, **A2**, **B1**, **B2**, **C1** and **C2** Expression levels of p-PI3Kp85, p-mTOR(Ser2448) and p-AKT(Ser473) in different group cells were detected by western blotting. The concentration of PcTx1 was 20 nM, the concentration of LY294002 was 1 µM, the concentration of Rapamycin was 0.5 nM, the concentration of MK-2206 was 0.1 µM, and all conducted at 24 h. Data were expressed as the mean ± SD, *n* = 3. n.s: no significance, *P < 0.05, **P < 0.01 and ***P < 0.001 all versus pH6.5 group

**Fig. 7:**
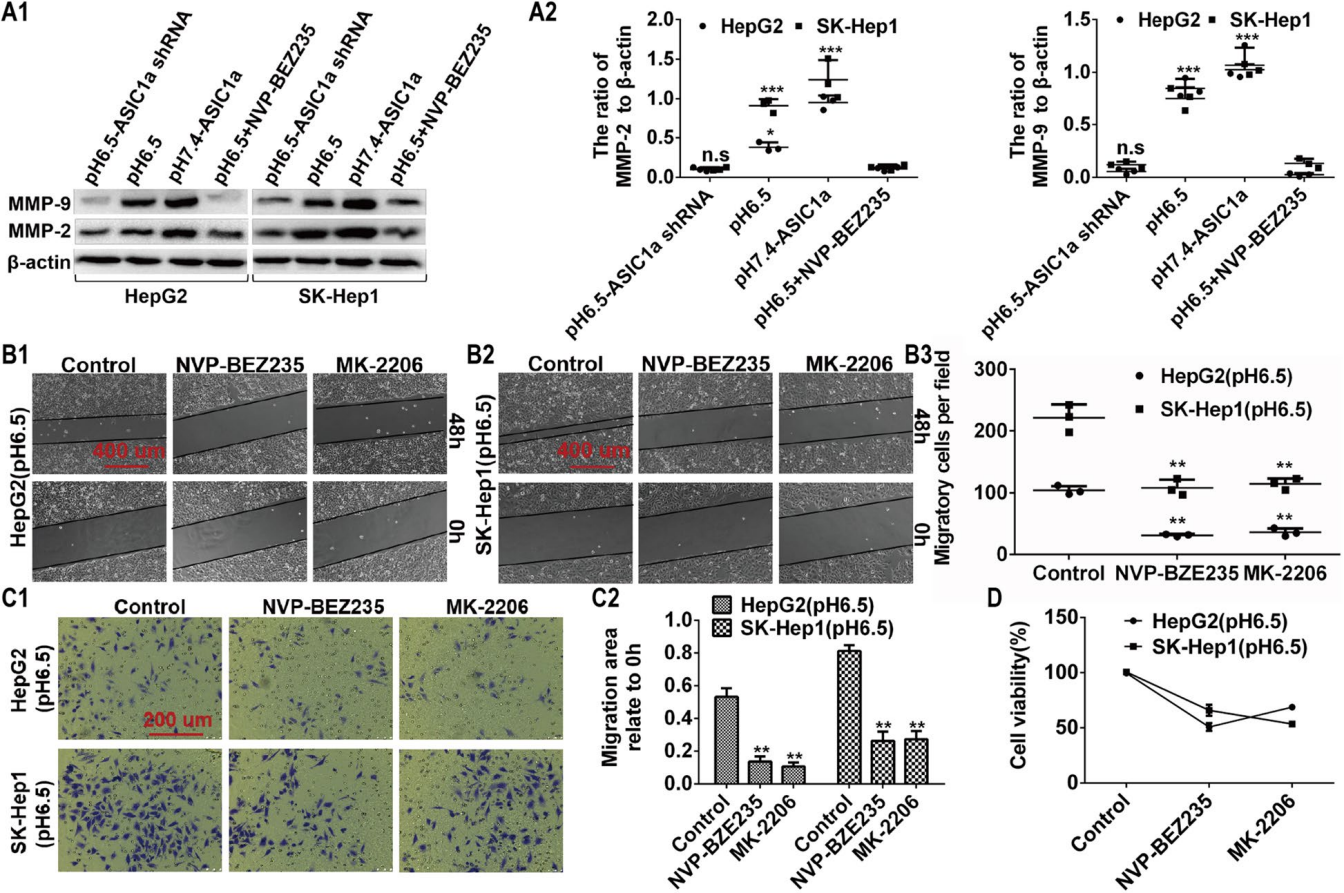
ASIC1α regulates the expression of MMP-2 and MMP-9 via the PI3K/AKT/mTOR pathway in HepG2 and SK-Hep1 cells. **A1**, **A2** and **A3** Expression levels of MMP-2 and MMP-9 in different group cells were detected by western blotting. The concentration of NVP-BEZ235 was 4 nM and the concentration of MK-2206 was 0.1 µM at 24 h. **B1**, **B2** and **B3** Migration abilities of different group cells were examined by wound healing assays. Representative images were taken at × 100 magnification. **C1** and **C2** Invasion abilities of different group cells were determined by transwell invasion assays. Representative images were taken at × 200 magnification. The concentration of NVP-BEZ235 was 4 nM and the concentration of MK-2206 was 0.1 µM at 24 h. **D** proliferation abilities of different group cells were determined by MTT assay. The concentration of NVP-BEZ235 was 4 nM and the concentration of MK-2206 was 0.1 µM at 24 h. Data were expressed as the mean ± SD, *n* = 3. n.s: no significance, *P < 0.05, **P < 0.01 and ***P < 0.001 all versus pH6.5 + NVP-BEZ235 group

**Fig. 8:**
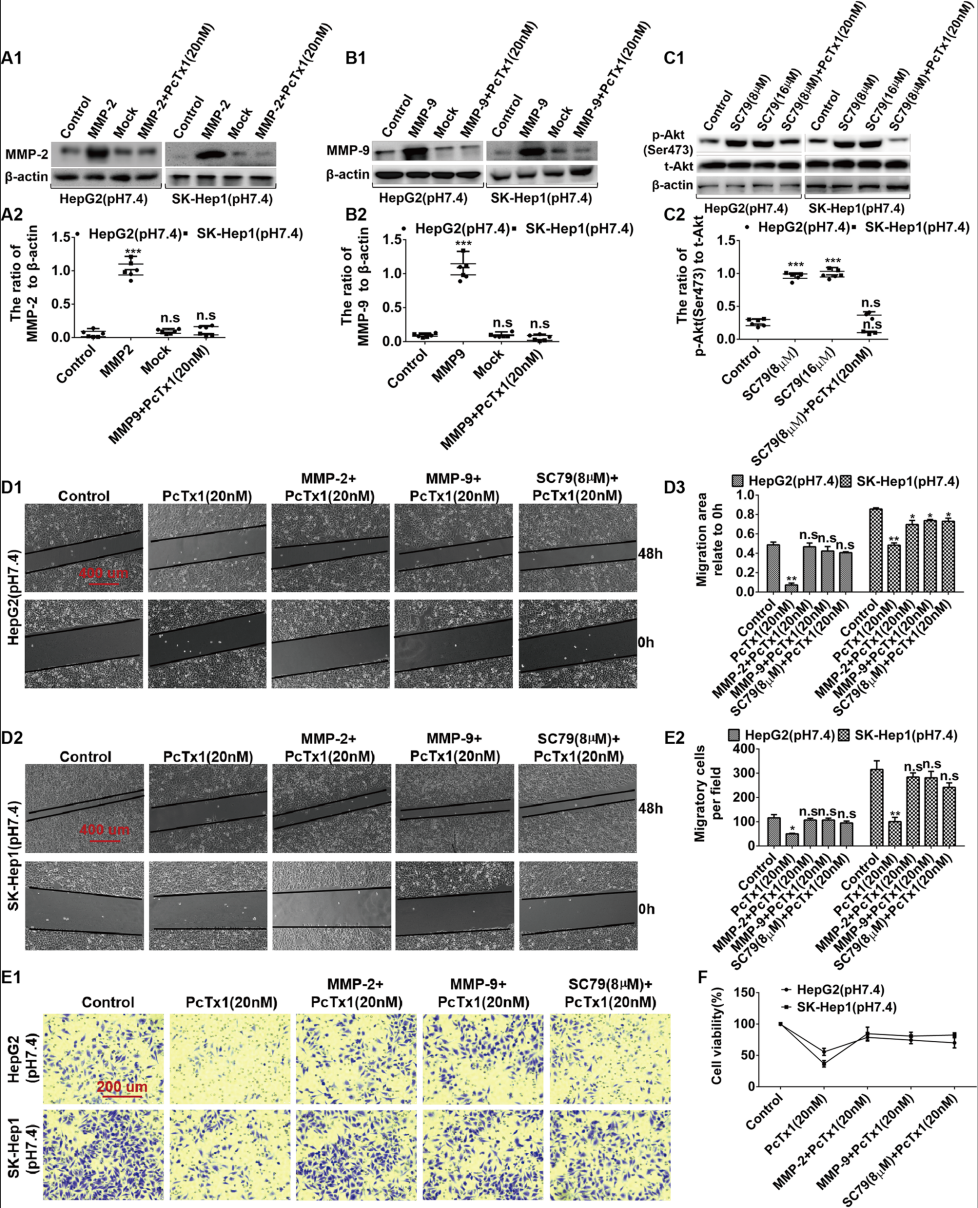
Overexpression of MMP-2/9 and activation of AKT can rescue the effect of reduced HepG2 and SK-Hep1 cells migration, invasion and proliferation caused by inhibiting ASIC1α. **A1**, **A2**, **B1**, **B2**, **C1** and **C2** Expression levels of MMP-2, MMP-9 and p-AKT(Ser473) in different group cells were detected by western blotting. The concentration of PcTx1 was 20 nM and the concentration of SC79 was 8, 16 µM at 24 h. **D1**, **D2** and **D3** Migration abilities of different group cells were examined by wound healing assays. Representative images were taken at × 100 magnification. The cells were treated with PcTx1 or SC79 for 6 h before performing wound healing assays. **E1** and **E2** Invasion abilities of different group cells were determined by transwell invasion assays. Representative images were taken at × 200 magnification. Invasion assays were conducted at 24 h. The cells were treated with PcTx1 or SC79 for 6 h before performing transwell invasion assays. **F** proliferation abilities of different group cells were determined by MTT assay. The concentration of PcTx1 was 20 nM and the concentration of SC79 was 8 µM at 24 h. Data were expressed as the mean ± SD, *n* = 3. n.s: no significance, *P < 0.05, **P < 0.01 and ***P < 0.001 all versus Control group

### ASIC1a can regulate aberrant activation of the PI3K/AKT/MTOR pathway

The PI3K/AKT/mTOR signaling pathway plays a key role in the development of tumors, but there has been little study of the link between ASIC1α and the pathway. As shown in Figs. 6A1, A2, B1, B2, C1, and C2, phosphorylation of PI3K, AKT, and mTOR was decreased in HepG2 and SK-Hep1 cells by ASIC1α shRNA, PcTX1 (20 nM), LY294002 (a broad-spectrum PI3K inhibitor), rapamycin (a specific inhibitor of mTOR), and MK-2206 (an AKT inhibitor). These data suggest that ASIC1a can regulate aberrant activation of the PI3K/AKT/MTOR pathway.

### ASIC1α regulates the level of MMP-2/9 via the PI3K/AKT/mTOR pathway in HepG2 and SK-Hep1 cells

ASIC1α can regulate MMP-2/9, and the PI3K/AKT signaling pathway can regulate ASIC1α. Thus, the question can be asked of whether ASIC1α regulates the expression of MMP-2/9 through the PI3K/AKT signaling pathway and thereby affects the migration, invasion, and proliferation in liver cancer cells. As shown in Figs. 7A1, A2, and A3, the expression levels of MMP-2/9 were significantly down-regulated in HepG2 (pH 6.5) cells treated with ASIC1α shRNA and NVP-BEZ235 (a dual PI3K/mTOR inhibitor). Similar results were obtained in SK-Hep1 cells. Then, as displayed in Figs. 7B1, B2, B3, C1, C2, and D, we observed that inactivation of the PI3K/AKT pathway by NVP-BEZ235 resulted in a decrease in HepG2 (pH 6.5) cell migration, invasion, and proliferation. Similar results were obtained with SK-Hep1 (pH6.5) cells.

### Overexpression of MMP-2/9 and activation of AKT can reverse the reduced migration, invasion, and proliferation of HepG2 and SK-Hep1 cells caused by inhibiting ASIC1α

To support our conclusion that MMP and PI3K/AKT mediate ASIC1α-regulated cell migration, invasion, and proliferation, we wished to demonstrate that MMP and AKT could reverse these effects by inhibiting ASIC1α. Thus, we used western blotting to determine the levels of MMP-2/9 and p-AKT(Ser473) after overexpression of MMP-2/9 and activation of AKT in HepG2 (pH 7.4) cells and SK-Hep1 (pH 7.4) cells. As illustrated in Figs. 8A1, A2, B1, B2, C1, and C2, the expression levels of MMP/9 and p-AKT(Ser473) were up-regulated by overexpression of MMP-2/9 and activation of AKT, as well as down-regulated by PcTx1 (20 nM) in HepG2 (pH 7.4) cells and SK-Hep1 (pH 7.4) cells. Then, we examined the migration, invasion, and proliferation abilities of HepG2 (pH 7.4) cells and SK-Hep1 (pH 7.4) cells with wound healing assay, transwell invasion assay, and MTT assay. Compared with the control group, the migration area relative to 0 h of PcTx1 (20 nM) cells was decreased (*P* < 0.01), whereas the migration area relative to 0 h of overexpression of MMP-2/9 and activation of AKT cells were neither significantly increased nor decreased (Figs. 8D1, D2 and D3). Similar results were obtained in studies of the invasion (Figs. 8E1 and E2) and proliferation (Fig. 8F) ability of HepG2 (pH 7.4) cells and SK-Hep1 (pH 7.4) cells. (Note: The cells were treated with PcTx1 or SC79 for 6 h before wound healing assays and transwell invasion assays were performed.) These data suggest that overexpression of MMP-2/9 and activation of AKT can reverse the reduced migration, invasion, and proliferation of HepG2 and SK-Hep1 cells caused by inhibiting ASIC1α.

## Discussion

The migration and invasion properties of liver cancer cells are key attributes for promoting liver cancer metastasis and malignant progression. The extracellular acidic microenvironment (about pH 6.5) is a common feature of most solid tumours and plays an important role in tumour growth, migration, and invasion [28–30]. ASIC1α promotes the migration, invasion, and proliferation of tumour cells as an acid sensor [9, 13]. However, the effect of ASIC1α in liver cancer cell migration, invasion, and proliferation, as well as specific molecular mechanisms and signal transduction pathways, remain unclear. In this study, we examined the expression of ASIC1α in HepG2 and SK-Hep1 cells cultured in various pH conditions and explored the effect of ASIC1α in the migration, invasion, and proliferation of liver cancer and its molecular mechanism through in vitro experiments. The results indicated that ASIC1α can up-regulate the expression of MMP-2/9 to enhance the migration, invasion, and proliferation of liver cancer cells via the PI3K/AKT/mTOR pathway.

Cancer is caused by genetic mutations and disruption of cellular homeostasis. These alterations create an extracellular environment called the tumour microenvironment, which includes surrounding blood vessels, immune cells, fibroblasts, bone marrow-derived inflammatory cells, various signaling molecules, and the extracellular matrix, and affects tumour development and metastasis. The acidic pH of the tumour microenvironment caused by altered tumour cell metabolism is characteristic of abnormal cell–cell interactions and disruption of homeostasis. In this state, tumour cells preferentially utilize glycolysis over oxidative phosphorylation as the primary means of energy release, an effect known as anaerobic glycolysis. The anaerobic glycolysis results in about a tenfold increase in the lactate load in the extracellular environment and the diffusion and transport of H + ions into the intercellular substance to avoid autoacidosis. These cellular responses also cause the pH of the tumour microenvironment to decrease and the overall environment to be acidic. In this study, we found that the expression levels of ASIC1α were significantly higher in liver cancer cells cultured in pH 6.5 than in pH 7.4, and the levels were especially higher than in the human normal liver cell line L-02. These results were consistent with those of previous studies [7, 8]. On the one hand, these findings indicate that ASIC1α is highly expressed in liver cancer tumour cells in normal physiological conditions and, on the other hand, up-regulation of ASIC1α likely is related to the occurrence and development of liver cancer.

However, it is not clear whether ASIC1α can significantly affect the migration, invasion, and proliferation of HepG2 and SK-Hep1 cells. To address this deficit, we first tested the effect of inhibiting the activity of ASIC1α (with PcTx1) on the liver cancer cells with the wound healing assay, transwell invasion assay, and MTT assay. The results revealed that in an acidic medium (pH 6.5), reducing the activity of ASIC1α enhanced the inhibition of migration, invasion, and proliferation of the HepG2 and SK-Hep1 cells. Also, silencing the ASIC1α gene enhanced these activities in the HepG2 and SK-Hep1 cells. Conversely, overexpression of the ASIC1α gene attenuated the inhibition of migration, invasion, and proliferation in the HepG2 and SK-Hep1 cells. These results suggest that activation of ASIC1α is a mechanism by which the metastasis and malignant progression of liver cancer is promoted. As illustrated in Fig. 5A, the pH 7.4-ASIC1α group had no effect on the expression level of MMPs compared with that of the pH 6.5 group, but in Fig. 7A, the pH 7.4-ASIC1α group up-regulated the expression level of MMP-2/9, which was due to the pH 6.5medium, used in Figs. 5A and 7A, the pH 7.4-ASIC1α group up-regulated the expression level of MMP-2/9, which was due to the pH 6.5medium, used in. not being prepared in the same batch, causing a slight difference in pH. In addition, HepG2 and SK-Hep1 cells were cultured in acidic (pH 6.5) medium to activate ASIC1α. There also were operational differences in each experiment, such as the precise control of timing, which also showed that ASIC1α is sensitive to acidic environment, and MMP-2/9 is regulated by ASIC1α.

MMP-2/9 is a major member of the matrix metalloproteinase family. The MMPs are upregulated in liver cancer cells, and inhibition of their expression inhibits the migration and invasion of liver cancer cells [31–35]. Inhibition of ASIC1α expression also can inhibit migration and invasion of liver cancer cells [9, 13]; so, whether ASIC1α and MMP-2/9 are correlated has interested us. Thus, we conducted the experiments illustrated in Fig. 5. First, the expression levels of MMP/9 in each group were analysed by western blotting; ASIC1a and MMP-2/9 were found positively correlated, i.e., inhibition of ASIC1α inhibited the expression of MMP-2/9. Next, we used wound healing assay, transwell invasion assay, and MTT assay to examine the migration, invasion, and proliferation of the liver cancer cells. On the one hand, ASIC1α mediated the migration, invasion, and proliferation of the cells. On the other hand, most importantly, decreased expression of MMP-2/9 inhibited the migration and invasion of liver cancer cells. All the above results established that ASIC1α positively regulates the expression of MMP-2/9 and promotes the migration, invasion, and proliferation of liver cancer cells.

The PI3K/AKT/mTOR pathway is abnormally activated in most tumour cells and mediates tumour cell growth, migration, invasion, and anti-apoptosis [36–40]. Also, ASIC1α can activate the PI3K/AKT/mTOR pathway and further mediate tumour cell resistance [13, 40]. In this work, we found that liver cancer cells cultured in pH 6.5 medium increased PI3K/AKT/mTOR activity, and expression of ASIC1α modulates the activity of this pathway. Inhibition of the PI3K/AKT/mTOR pathway reduced the expression level of MMP-2/9 and inhibited the migration, invasion, and proliferation of liver cancer cells. Thus, ASIC1α can upregulate the expression level of MMP-2/9 by activating the PI3K/AKT/mTOR signaling pathway to promote the migration, invasion. and proliferation of liver cancer cells. This biological behaviour contributes to the metastasis and malignant progression of liver cancer cells.

Since the two compounds have different dose relationships that may lead to different results, we analyzed the dose relationship of the two compounds, the Akt activator, SC79, and the ASIC1α inhibitor, PcTx1, based on dose–response results at the beginning the study and finally determined a combination with the best dose–response results. Activators affect protein activity, such as acetylation, phosphorylation, and ubiquitination activation, whereas gene amplification technology directly increases protein expression. In this study, we needed to examine the reversal role of Akt protein activity (phosphorylation) on ASIC1α inhibition. Therefore, we used the Akt activator SC79 without the gene amplification technique used in the MMP group.

Due to limitations of laboratory conditions, we selected target cell lines based on the existing cells in the laboratory, not by detecting the expression of ASIC1α on all liver cancer cells. We admit that this is a limitation of our study, which we will correct in future research. In addition, drug resistance of liver cancer cells is an extremely important factor in promoting tumor progression. We will explore the relationship between ASIC1α and the drug resistance of liver cancer cells and its molecular mechanism in the next study.

## Conclusions

Overexpression of the acid-sensitive ion channel 1α (ASIC1α) affects the molecular mechanisms responsible for the migration, invasion, and proliferation of liver cancer cells. This action is achieved through modulation of the MMP-2/9/PI3K/AKT signaling pathway in the extracellular acidic microenvironment. Appreciation of this mechanism may be useful in the diagnosis and treatment of liver cancer.

## Supplementary Information

**Additional file 1.** 

## Data Availability

The datasets used and/or analysed during the current study are available from the corresponding author on reasonable request.
