# Supplementary material for: ASIC1α up-regulates MMP-2/9 expression to enhance mobility and proliferation of liver cancer cells via the PI3K/AKT/mTOR pathway
Source: BMC Cancer. 2022 Jul 16;22:778. doi: 10.1186/s12885-022-09874-w (PMC9287982; doi:10.1186/s12885-022-09874-w)

# ASIC1α up-regulates MMP-2/9 expression to enhance mobility and proliferation of liver cancer cells via the PI3K/AKT/mTOR pathway

**Supplementary Information file**

**Fig 1A1**

**ASIC1α (HepG2+L-02)**

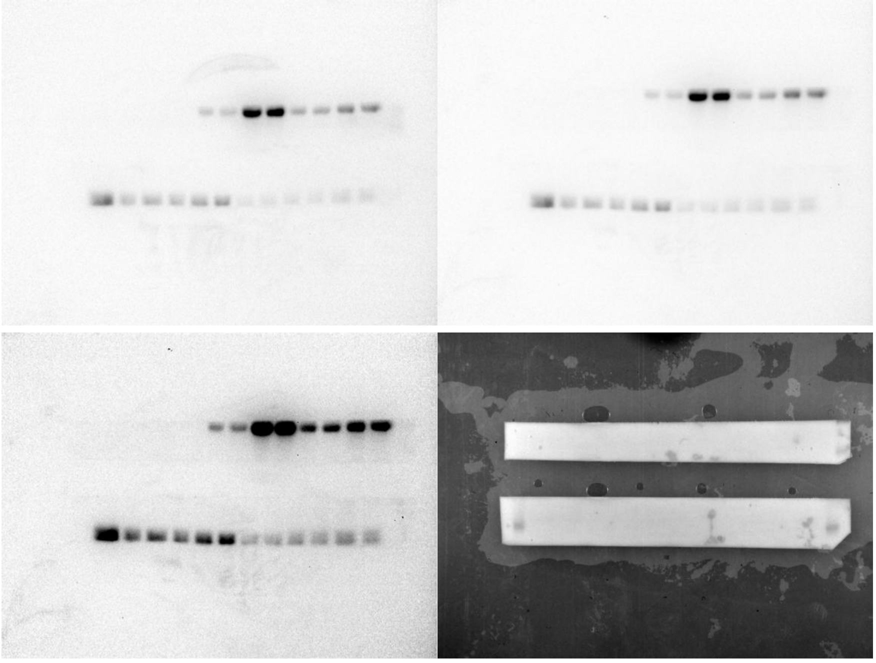

**β-actin (HepG2+L-02)**

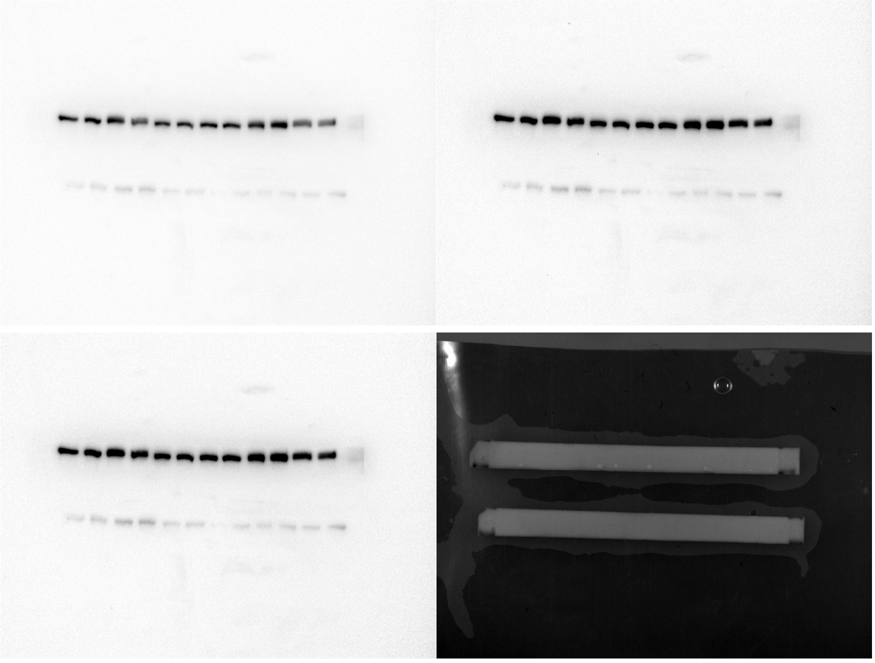

**ASIC1α (SK-Hep1+L-02)**

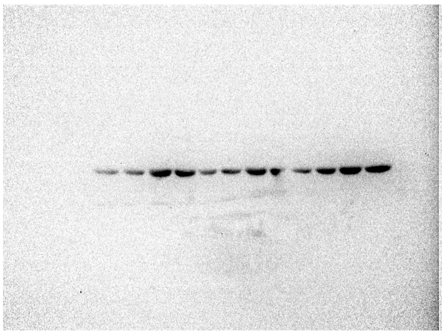
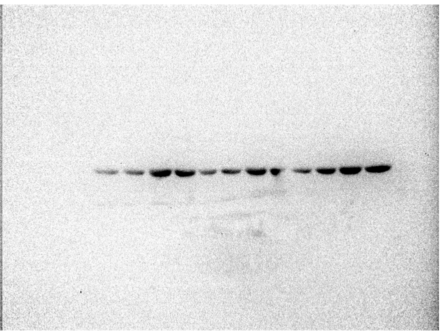

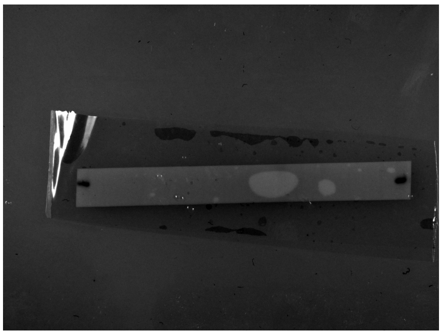

**β-actin (SK-Hep1+L-02)**

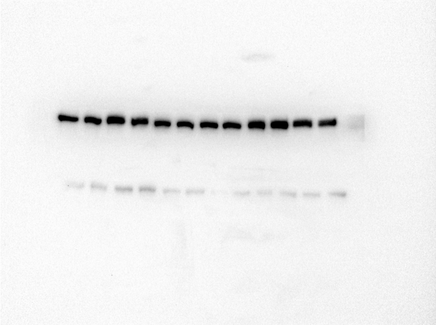
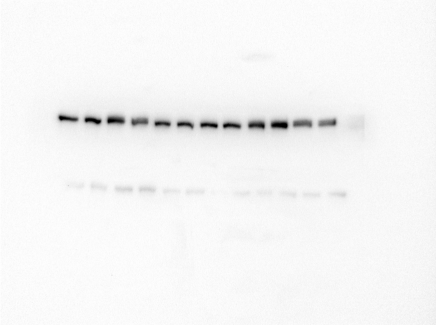

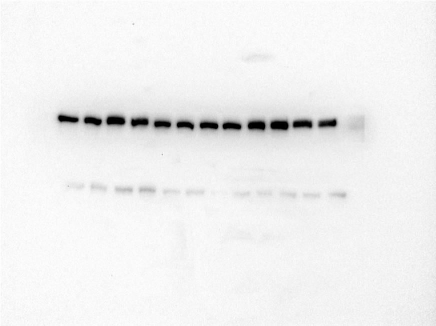
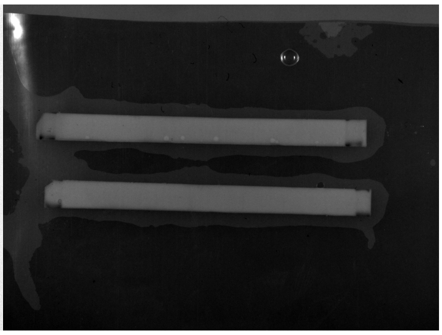

**Fig 2A1**

**ASIC1α (HepG2+L-02)**

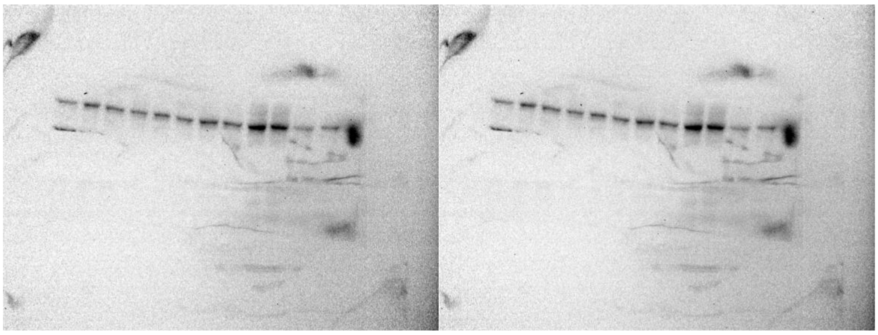

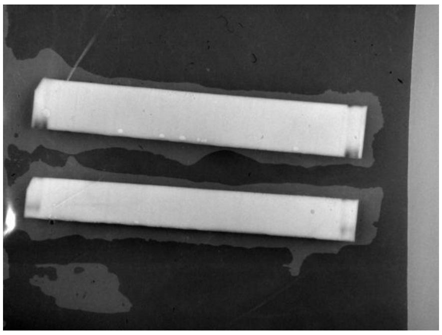

**β-actin (HepG2+L-02)**

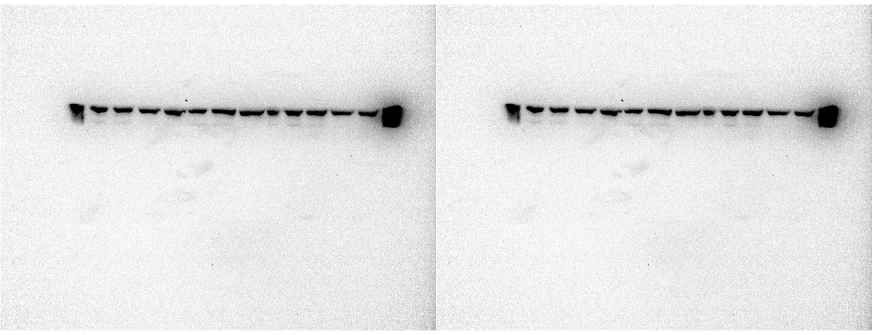

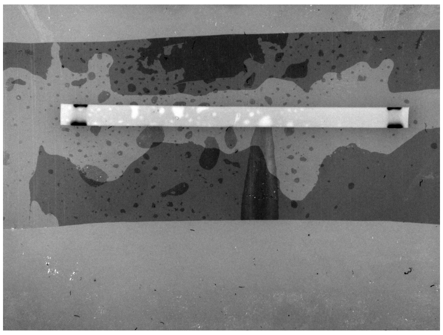

**ASIC1α (SK-Hep1+L-02)**

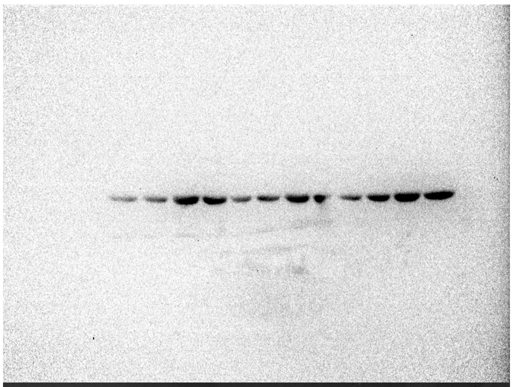

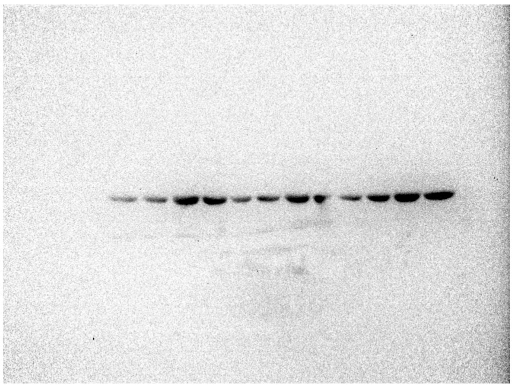

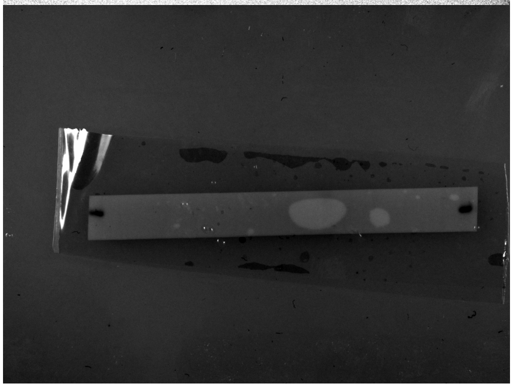

**β-actin (SK-Hep1+L-02)**

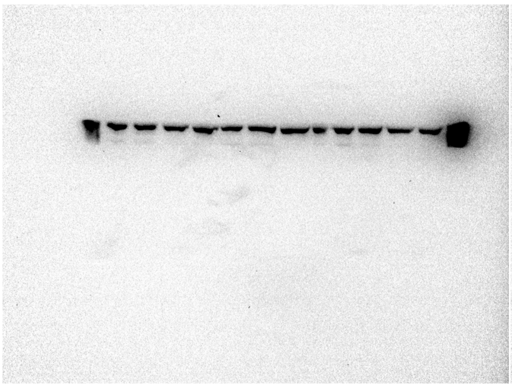

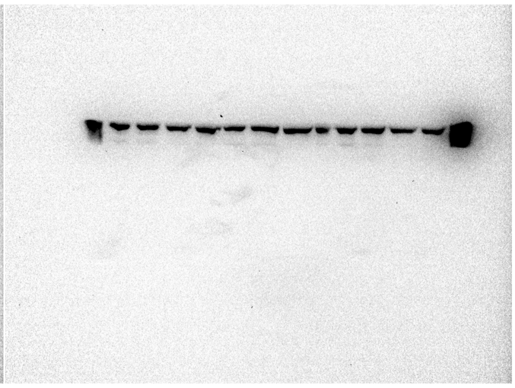

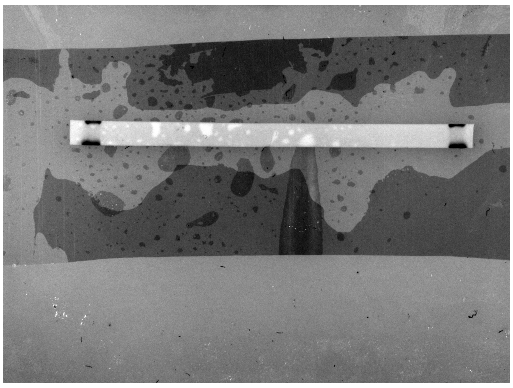

**Fig 3A1**

**ASIC1α (HepG2+L-02)**

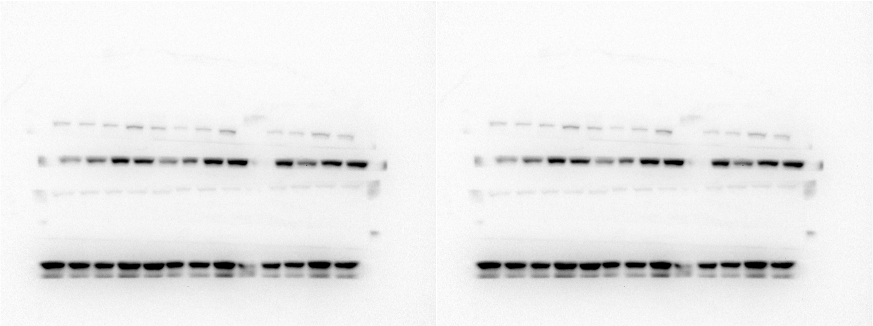

**β-actin (HepG2+L-02)**

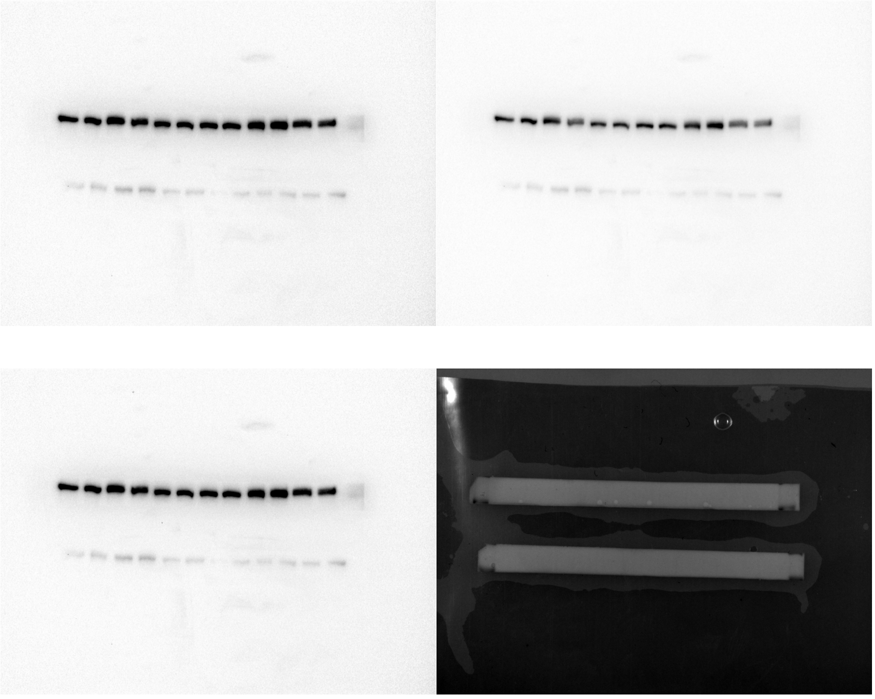

**ASIC1α (SK-Hep1+L-02)**

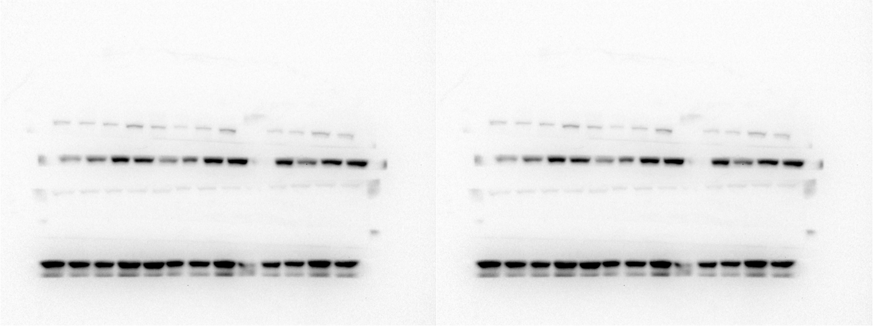

**β-actin (SK-Hep1+L-02)**

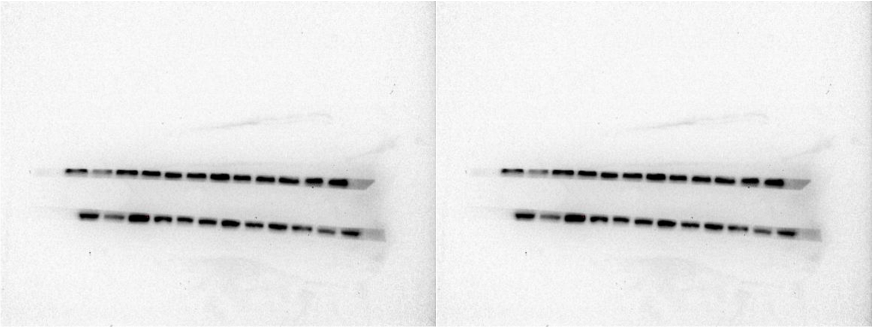

**Fig 4A1**

**ASIC1α (HepG2+L-02)**

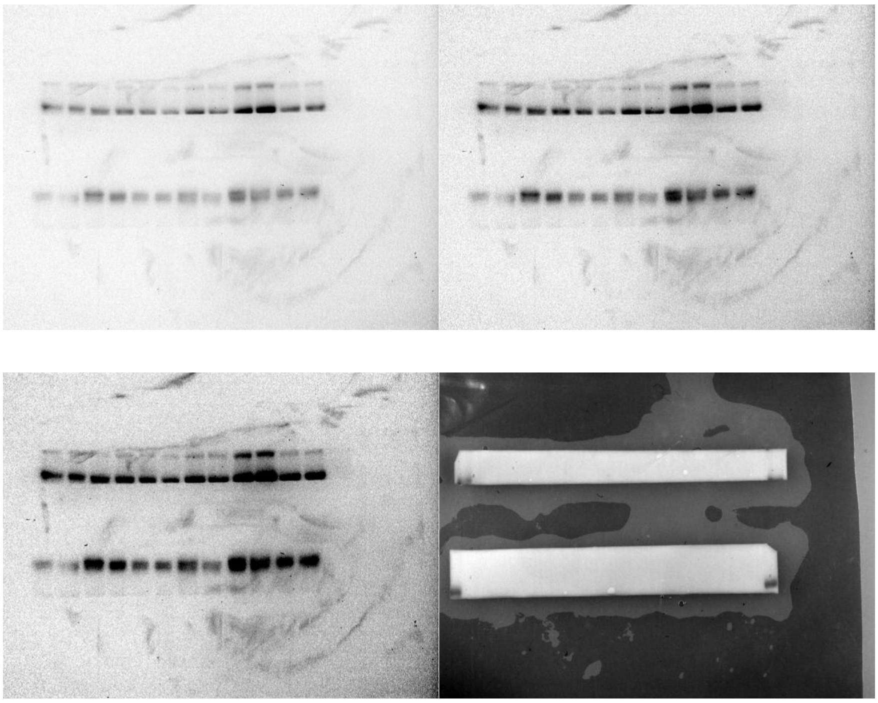

**β-actin (HepG2+L-02)**

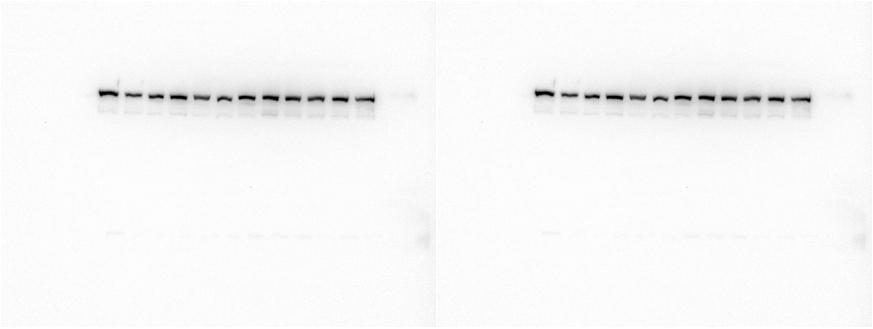

**ASIC1α (SK-Hep1+L-02)**

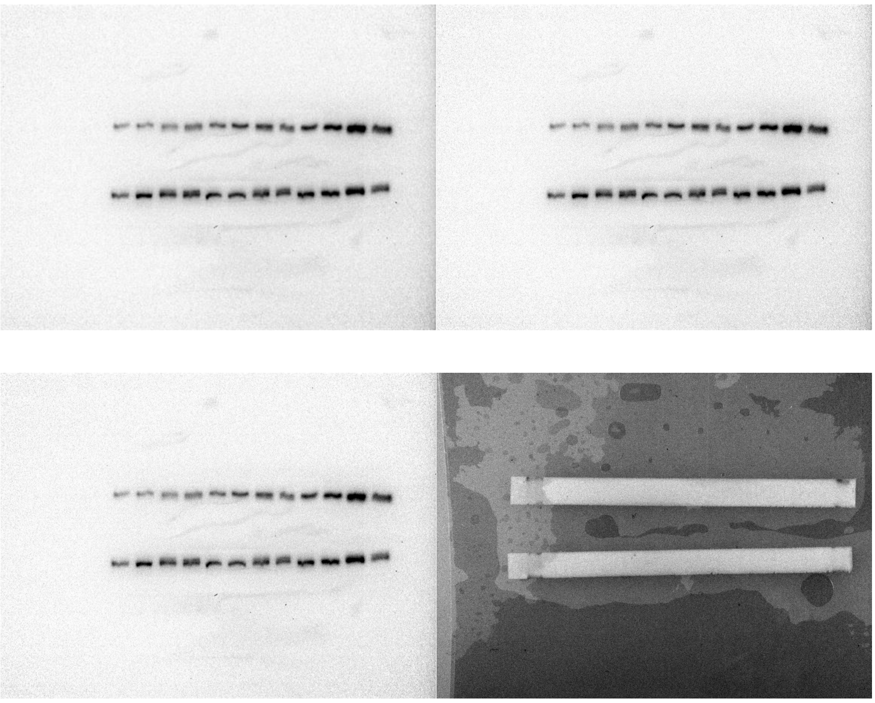

**β-actin (SK-Hep1+L-02)**

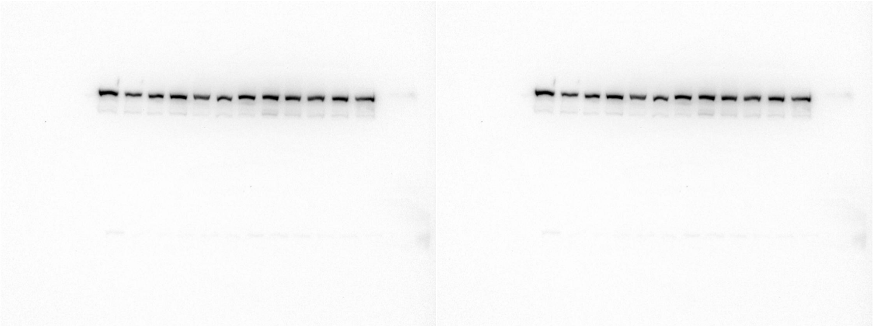

**Fig 5A1**

**MMP-9 (HepG2+L-02)**

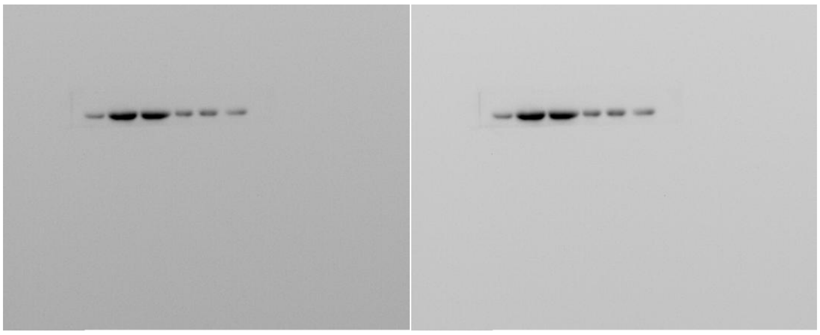

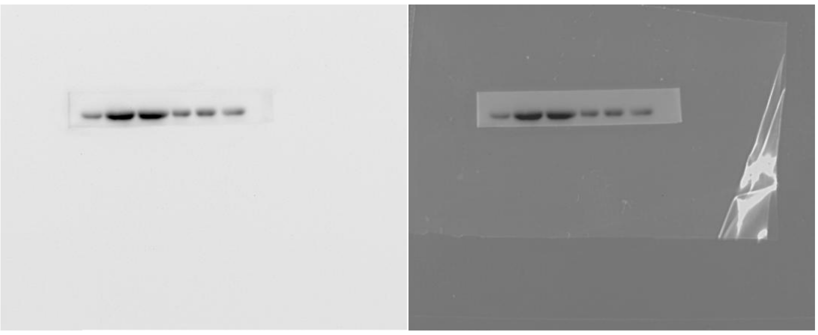

**MMP-2 (HepG2+L-02)**

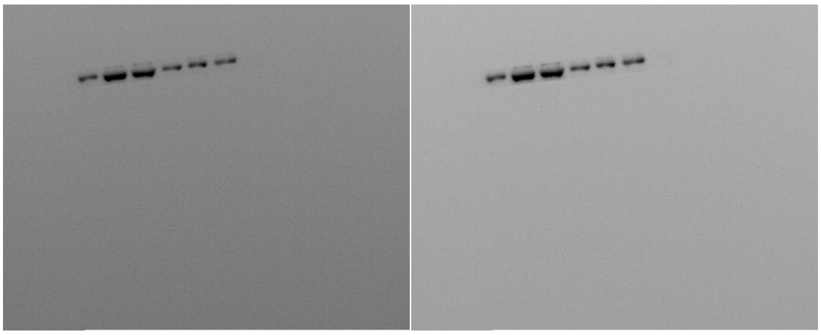

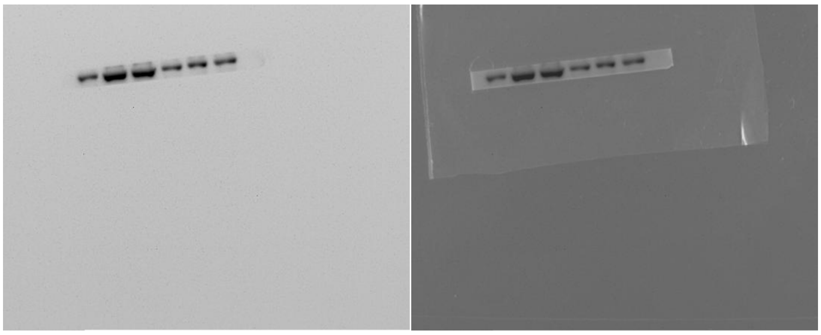

**β-actin (HepG2+L-02)**

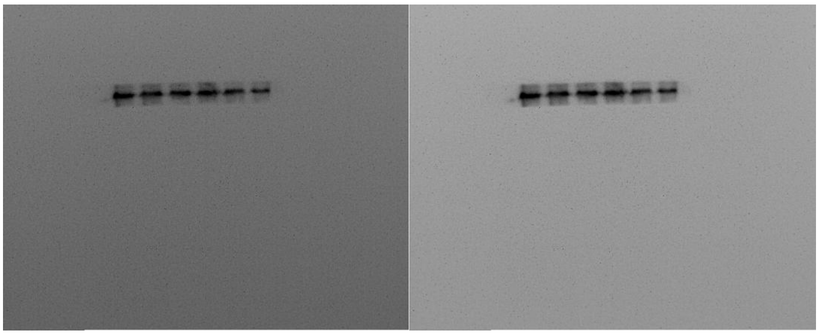

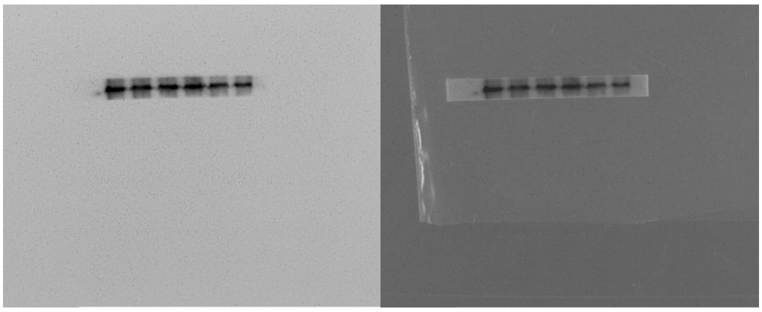

**MMP-9 (SK-Hep1+L-02)**

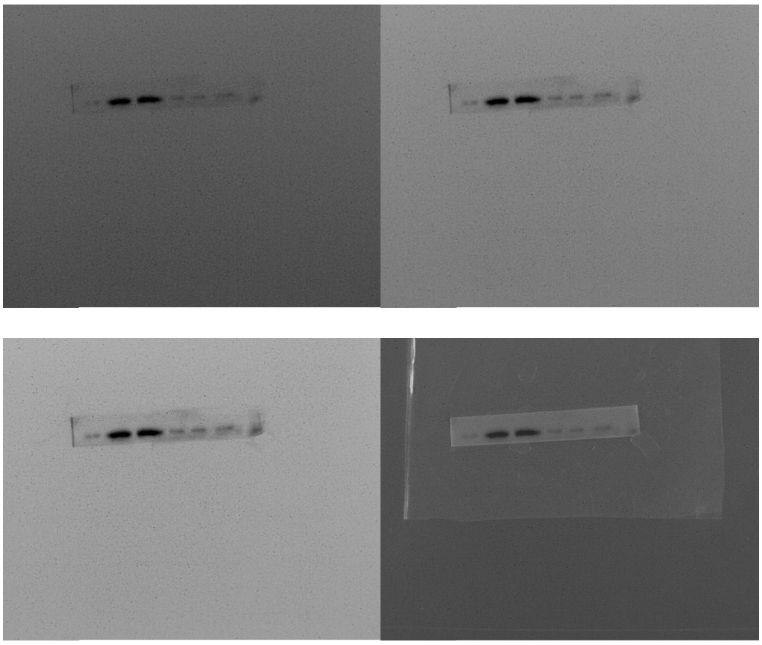

**MMP-2 (SK-Hep1+L-02)**

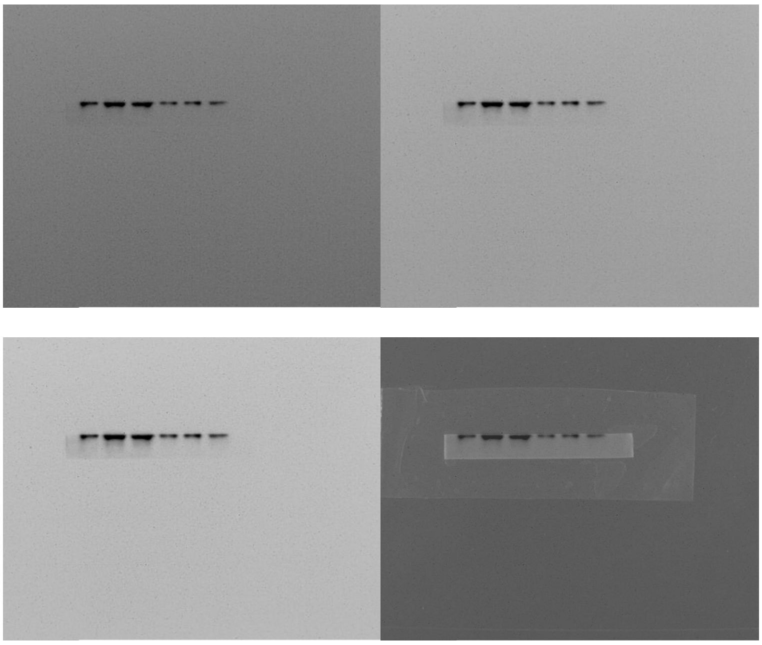

**β-actin (SK-Hep1+L-02)**

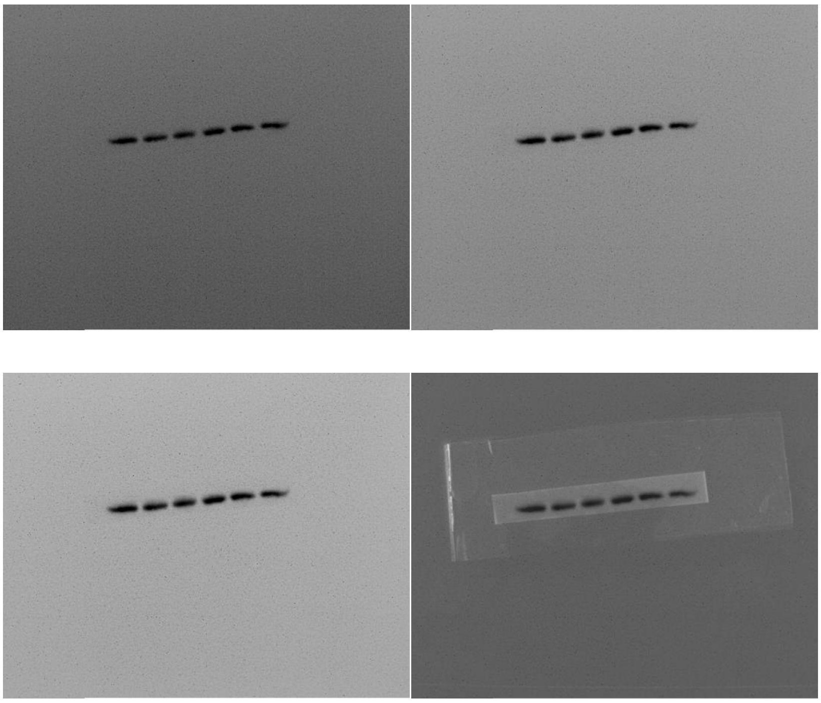

**Fig 6A1**

**p-PI3Kp85 (HepG2+L-02)**

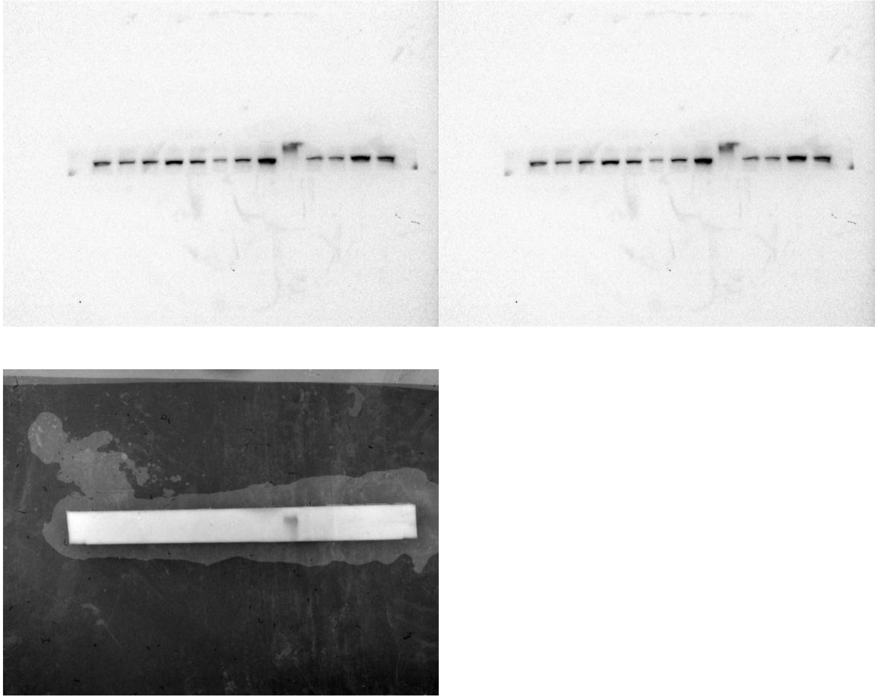

**t-PI3Kp85 (HepG2+L-02)**

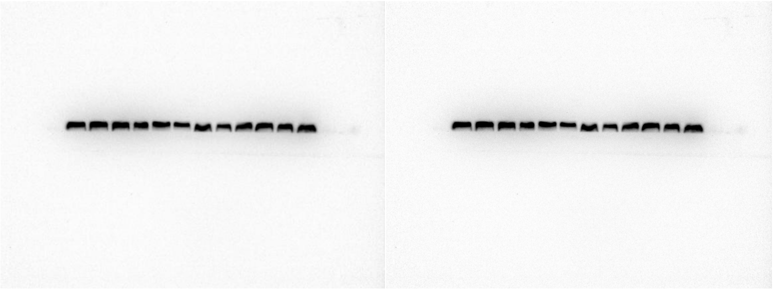

**β-actin (HepG2+L-02)**

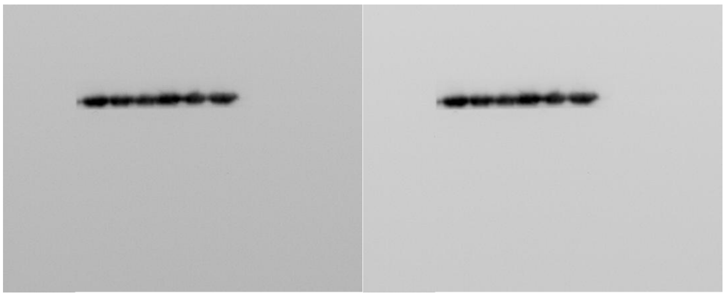

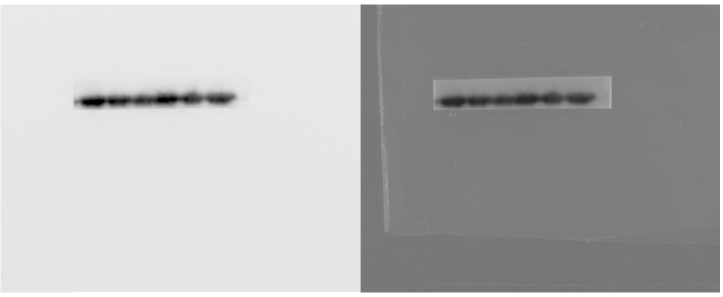

**p-PI3Kp85 (SK-Hep1+L-02)**

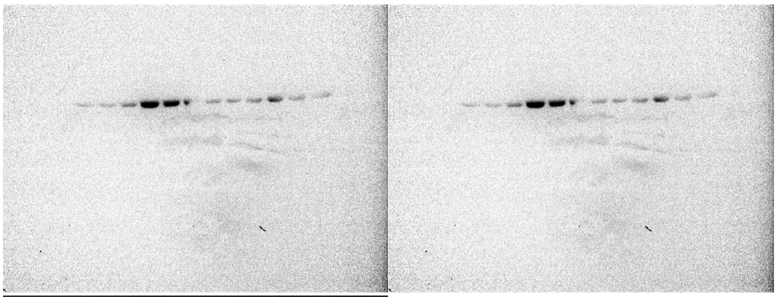

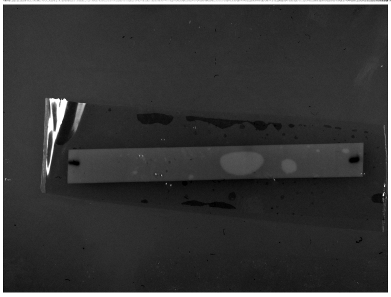

**t-PI3Kp85 (SK-Hep1+L-02)**

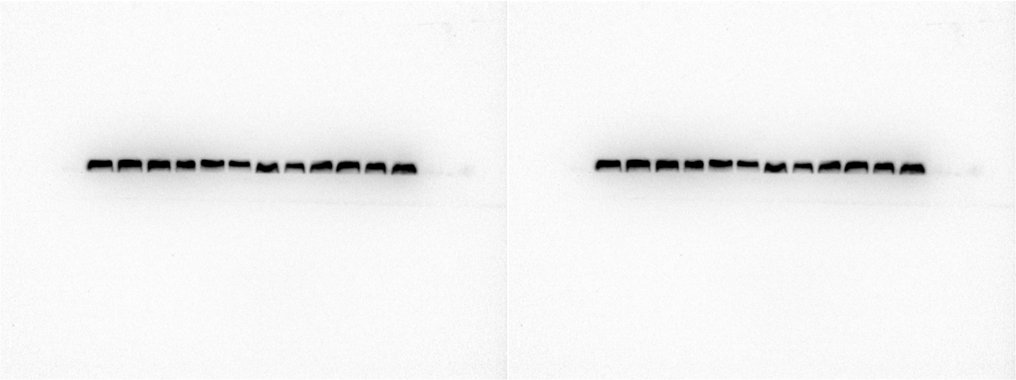

**β-actin (SK-Hep1+L-02)**

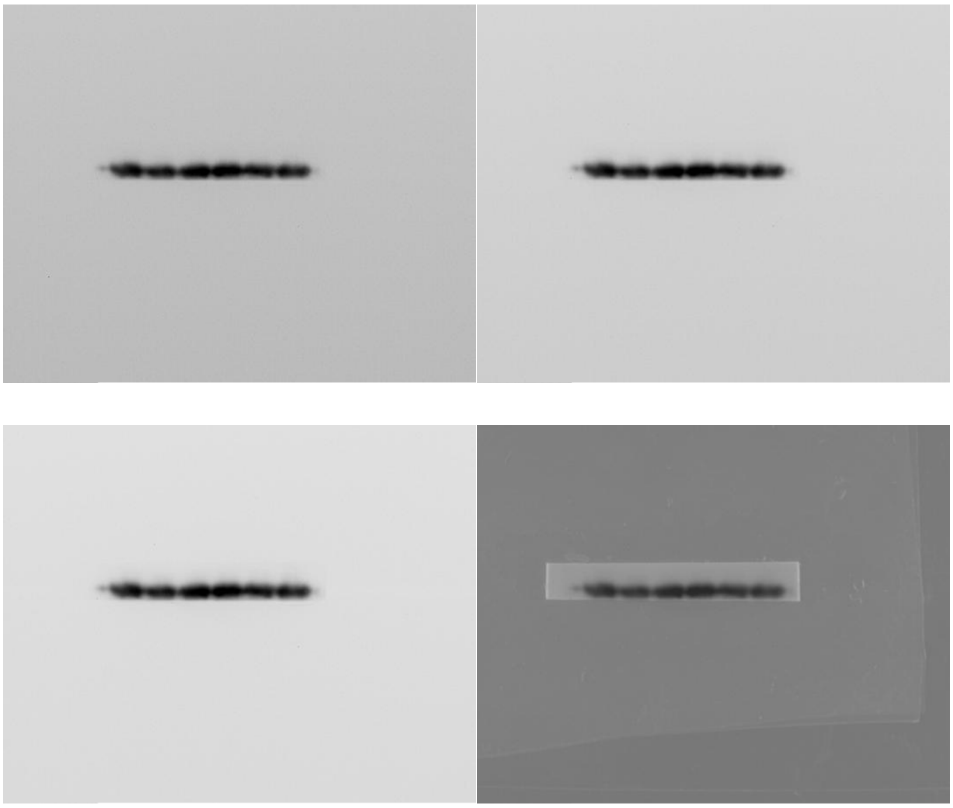

**Fig 6B1**

**p-mTOR(Ser2448) (HepG2+L-02)**

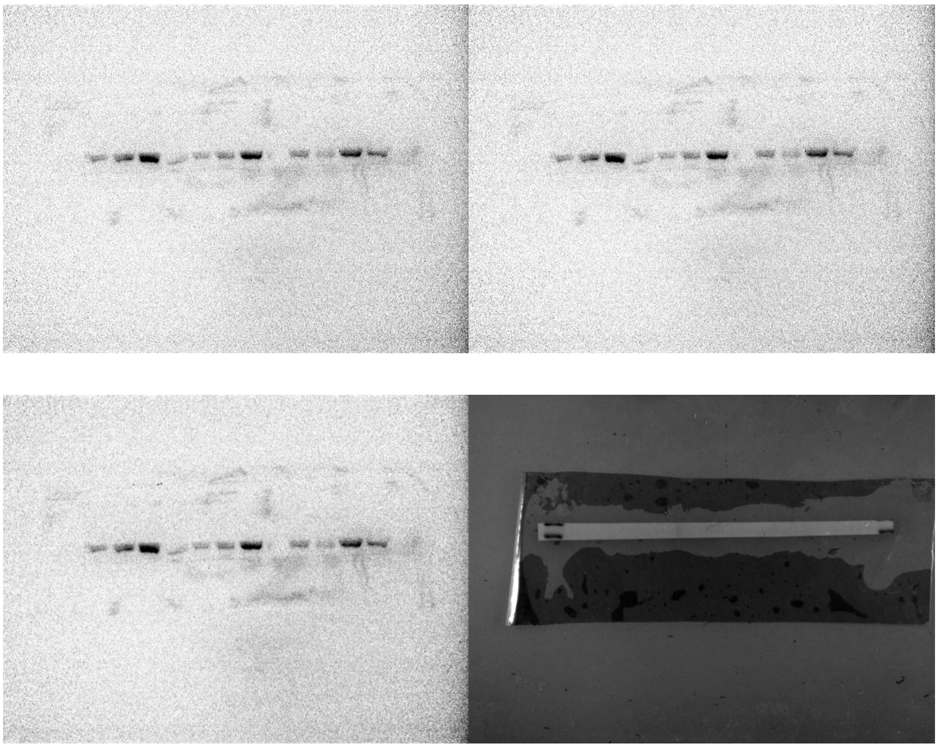

**t-mTOR (HepG2+L-02)**

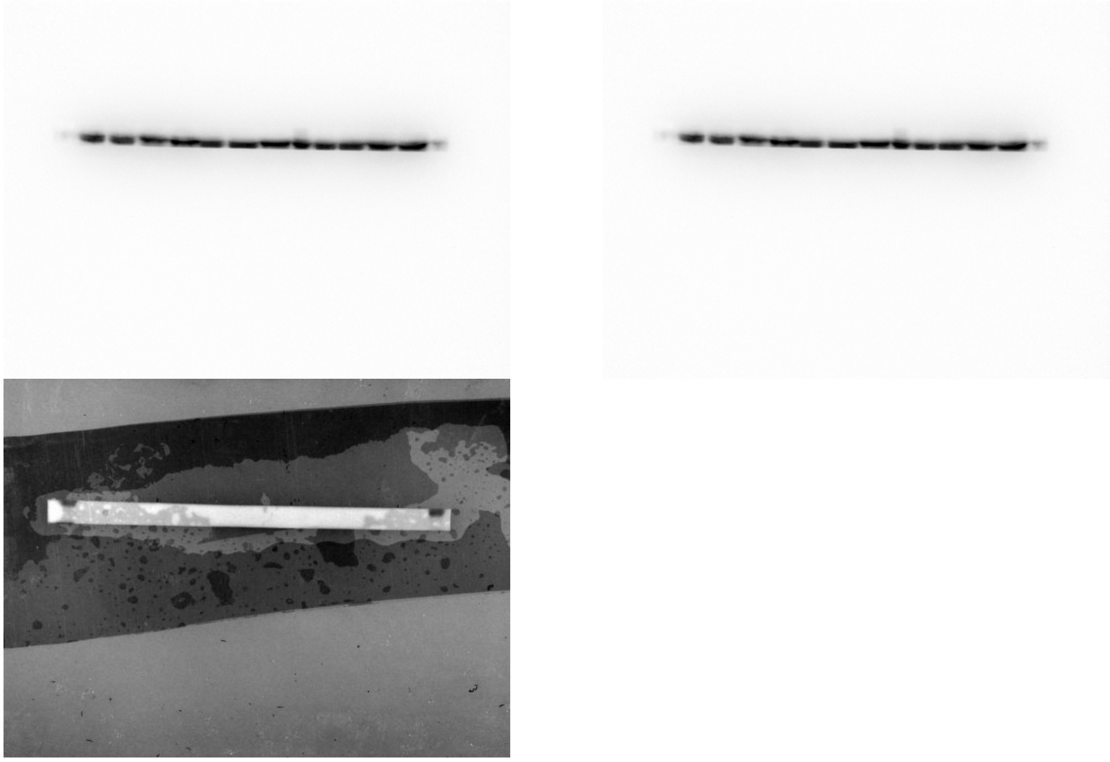

**β-actin (HepG2+L-02)**

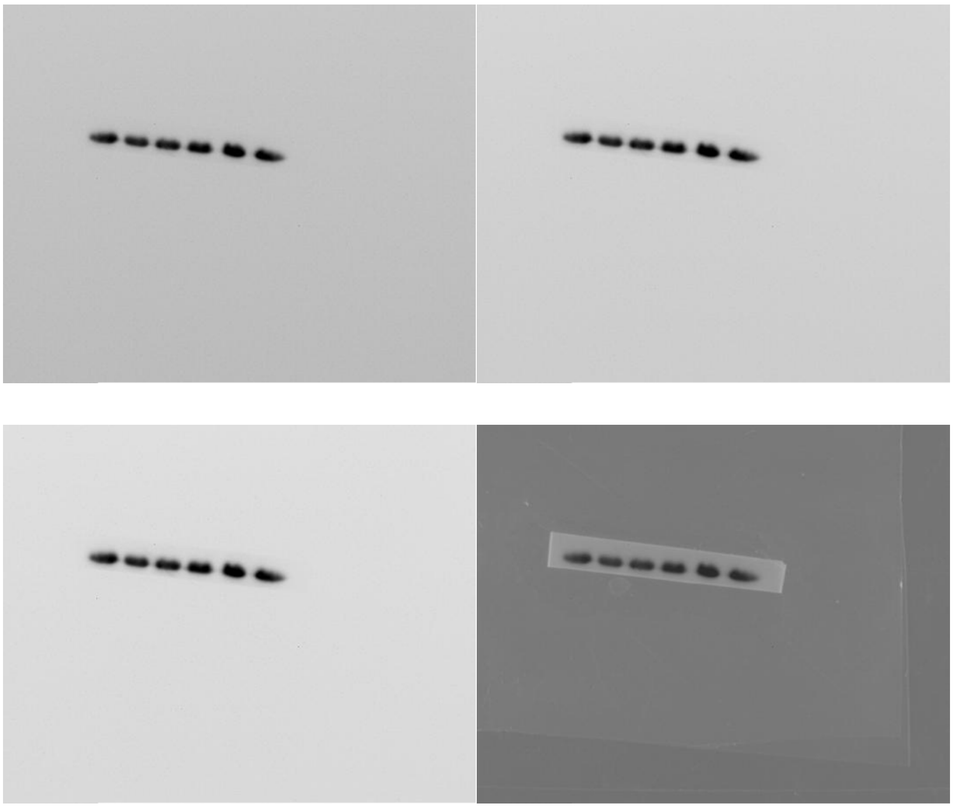

**p-mTOR(Ser2448) (SK-Hep1+L-02)**

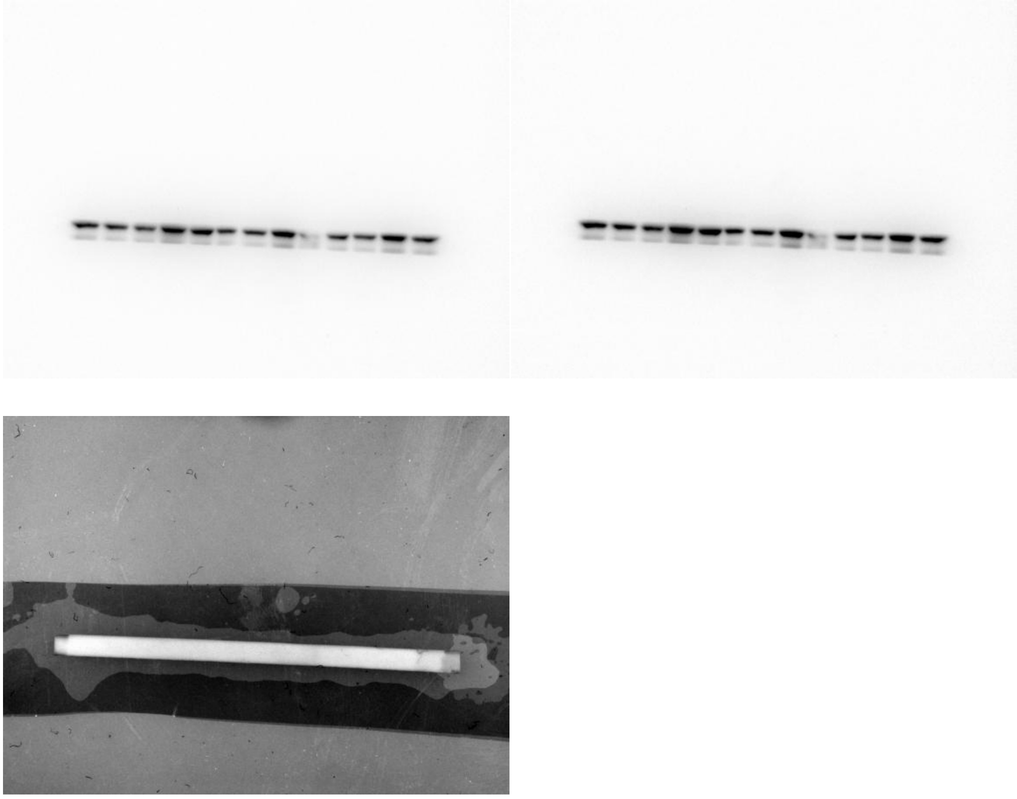

**t-mTOR (SK-Hep1+L-02)**

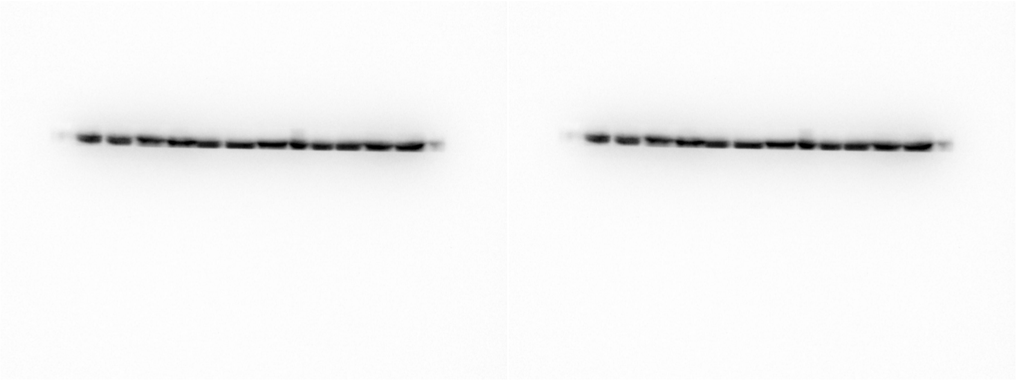

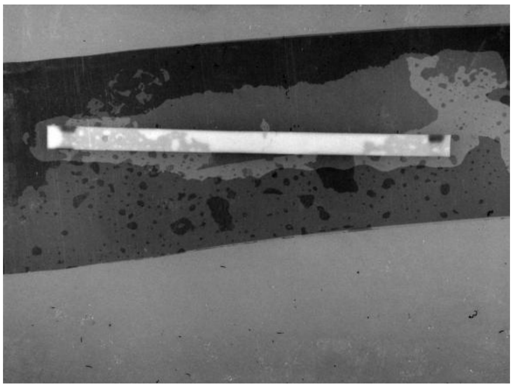

**β-actin (SK-Hep1+L-02)**

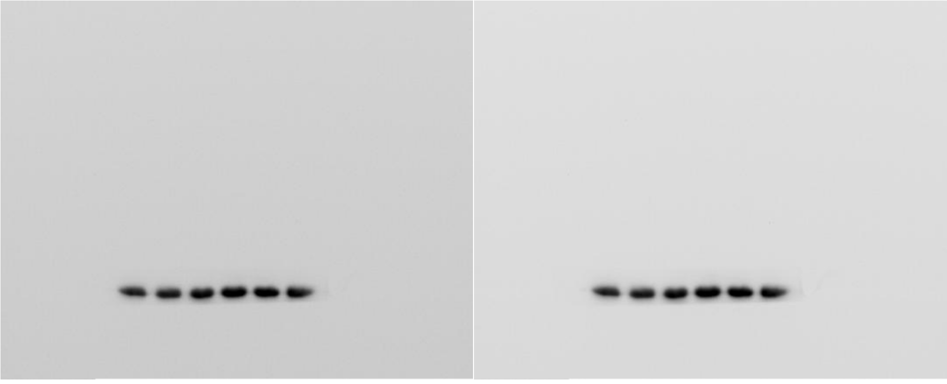

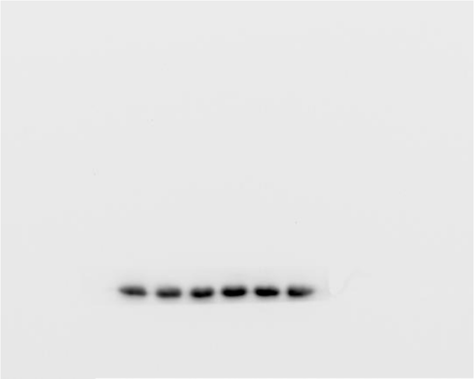

**Fig 6C1**

**p-Akt(Ser473) (HepG2+L-02)**

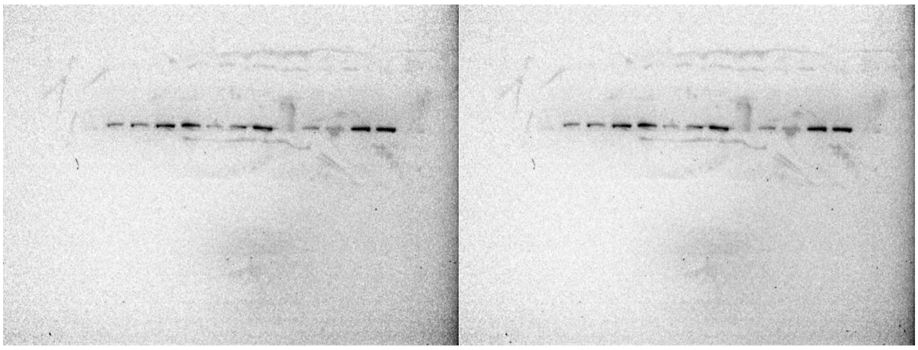

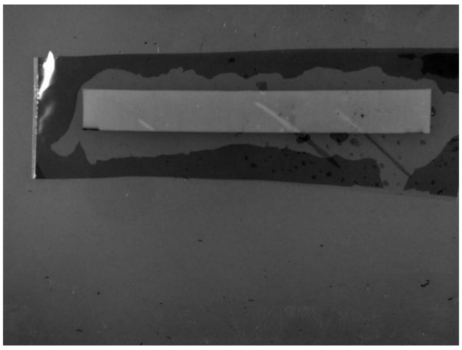

**t-Akt (HepG2+L-02)**

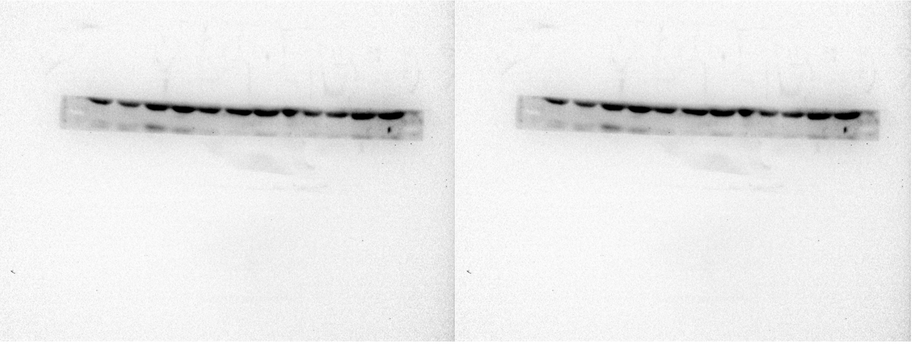

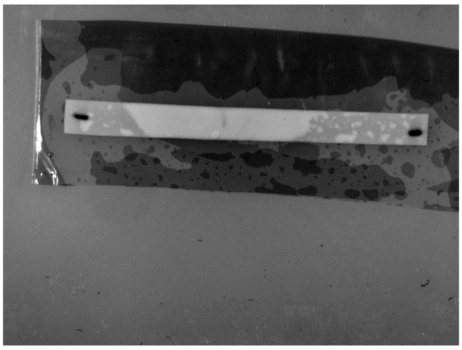

**β-actin (HepG2+L-02)**

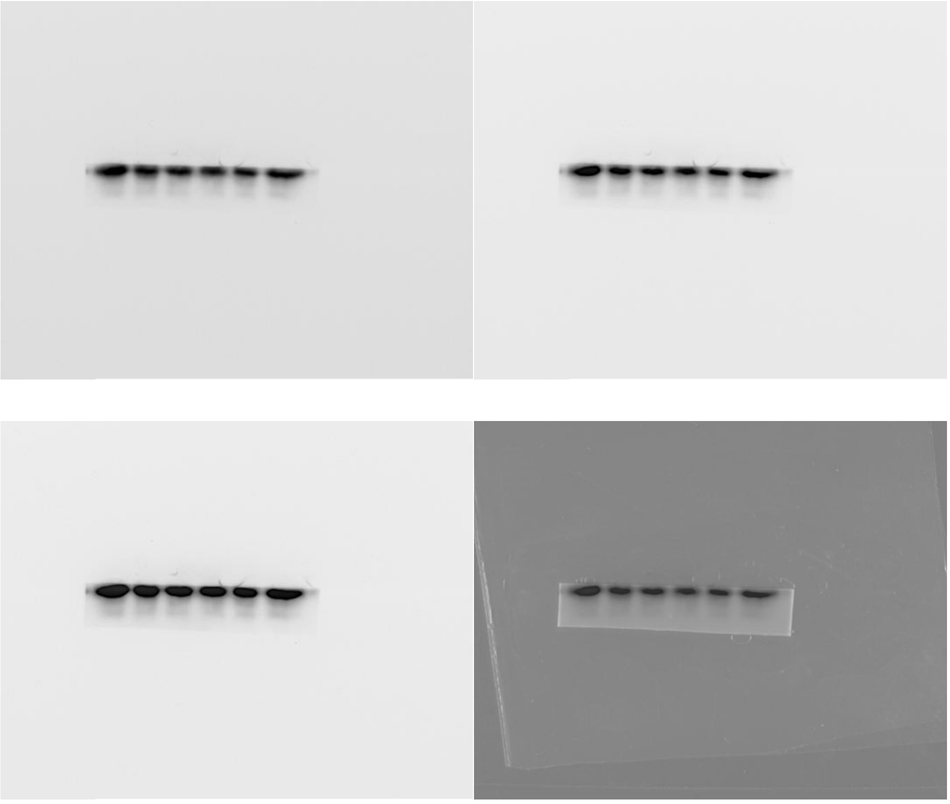

**p-Akt(Ser473) (SK-Hep1+L-02)**

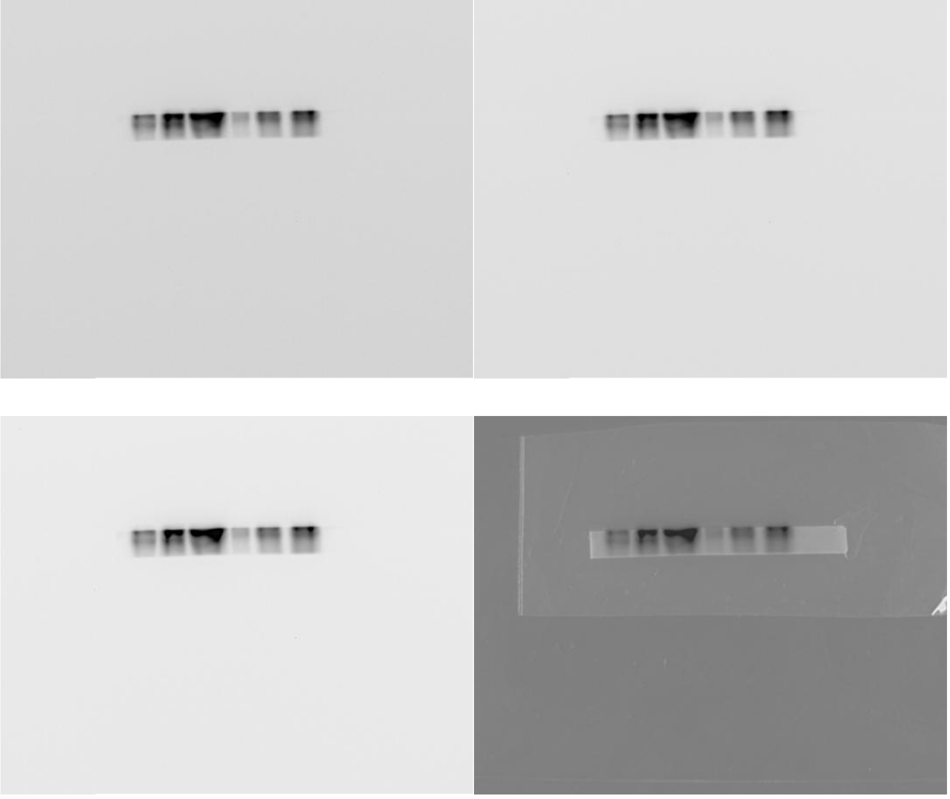

**t-Akt (SK-Hep1+L-02)**

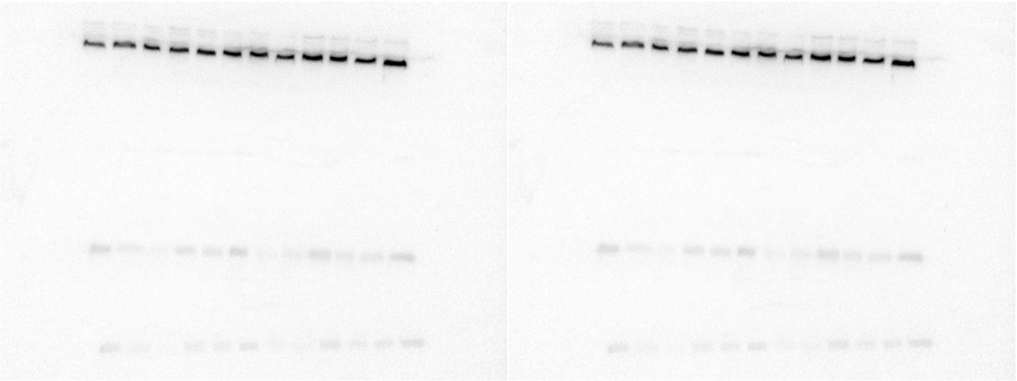

**β-actin (SK-Hep1+L-02)**

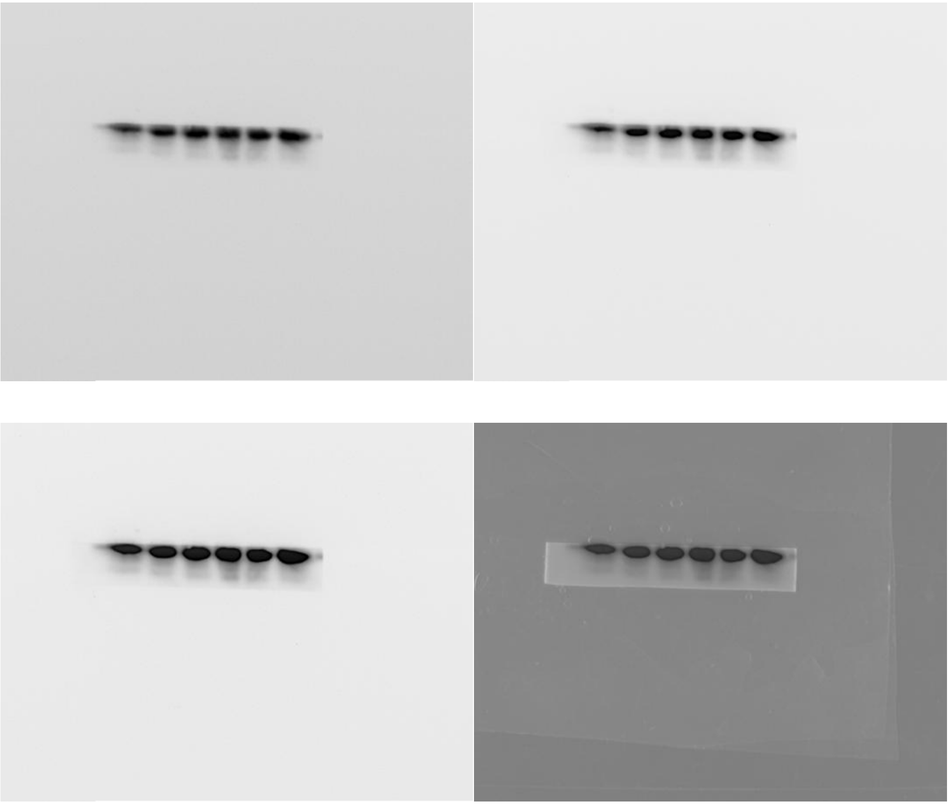

**Fig 7A1**

**MMP-9 (HepG2)**

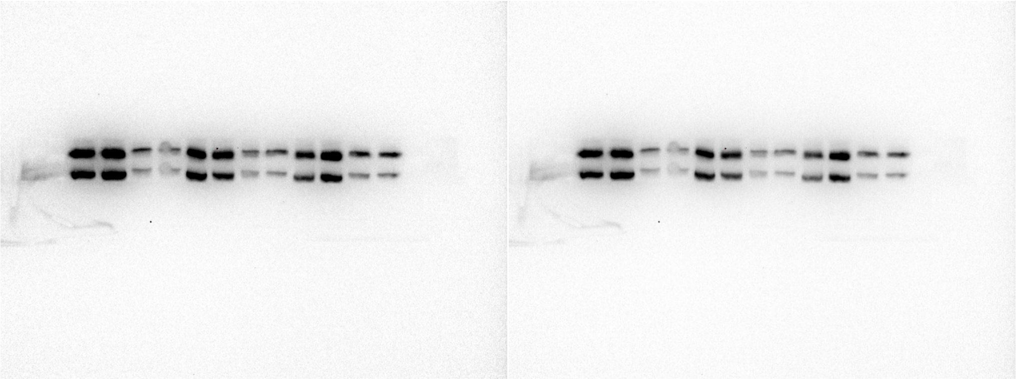

**MMP-2 (HepG2)**

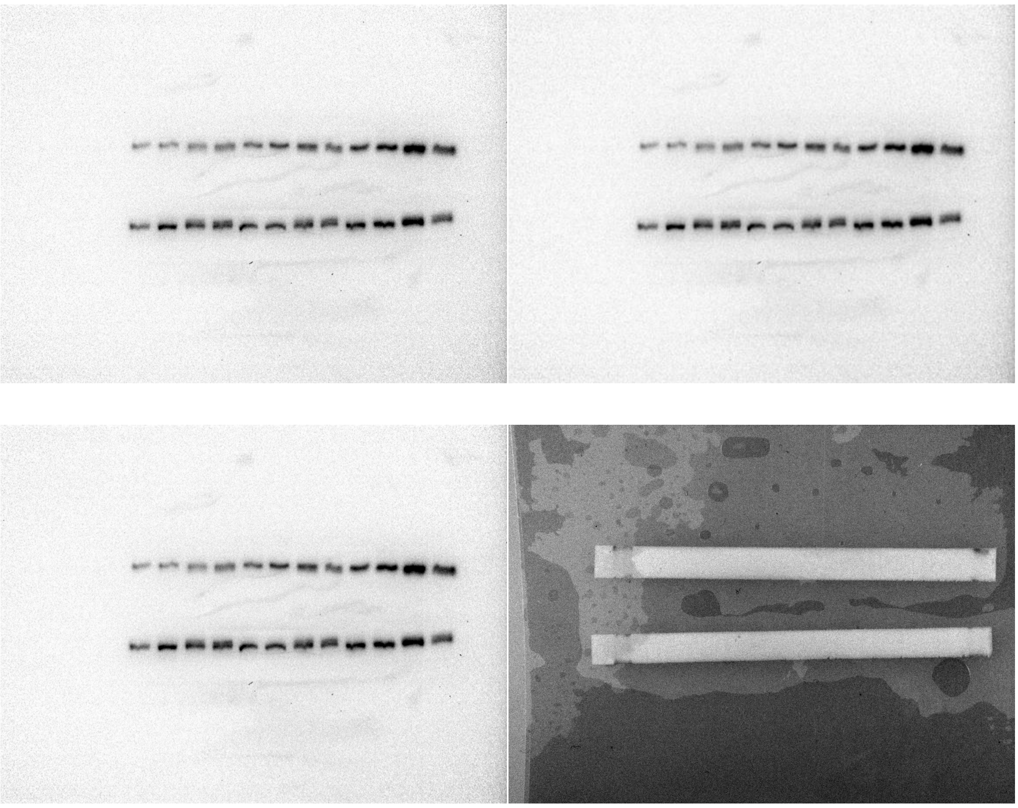

**β-actin (HepG2)**

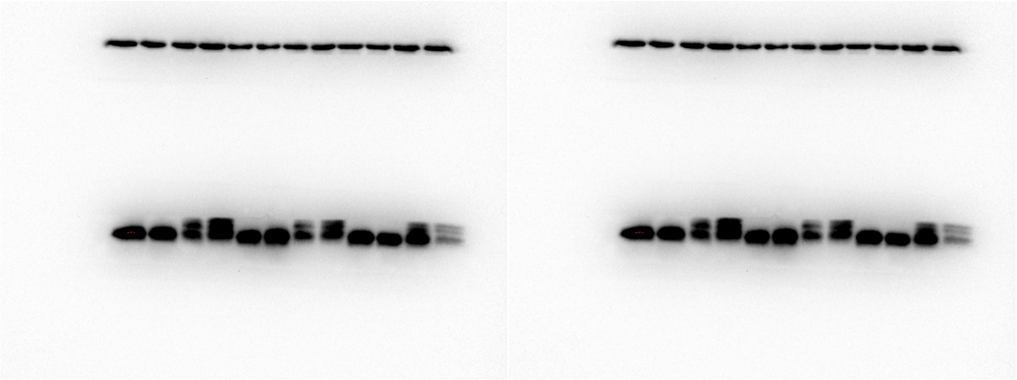

**MMP-9 (SK-Hep1)**

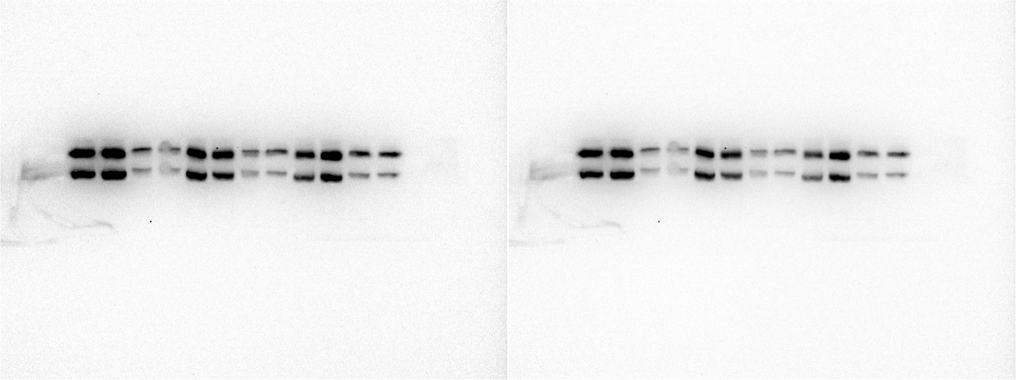

**MMP-2 (SK-Hep1)**

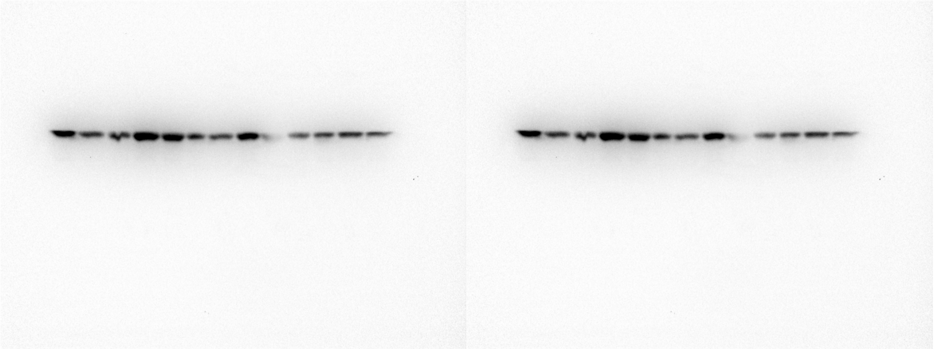

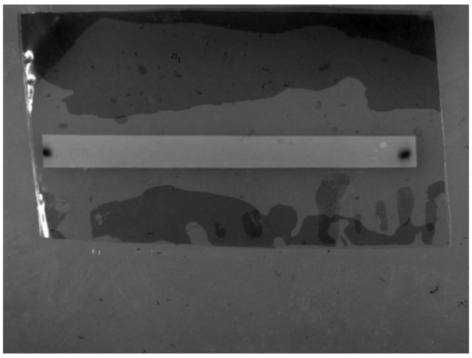

**β-actin (SK-Hep1)**

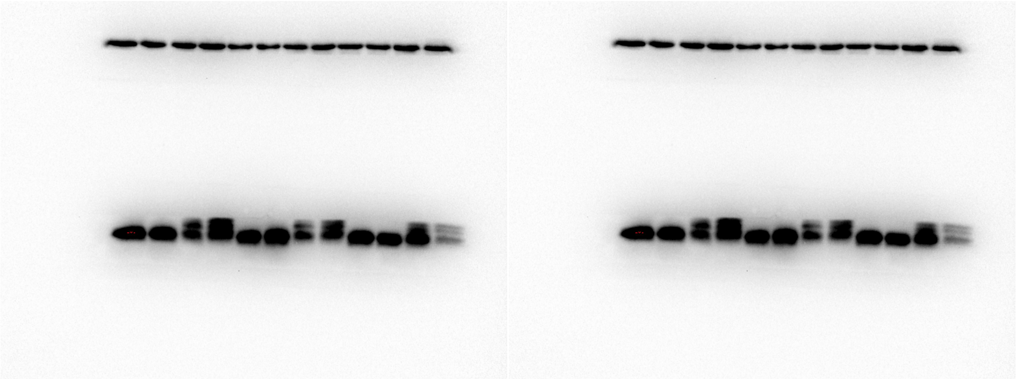

**Fig 8A1**

**MMP-2 (HepG2)**

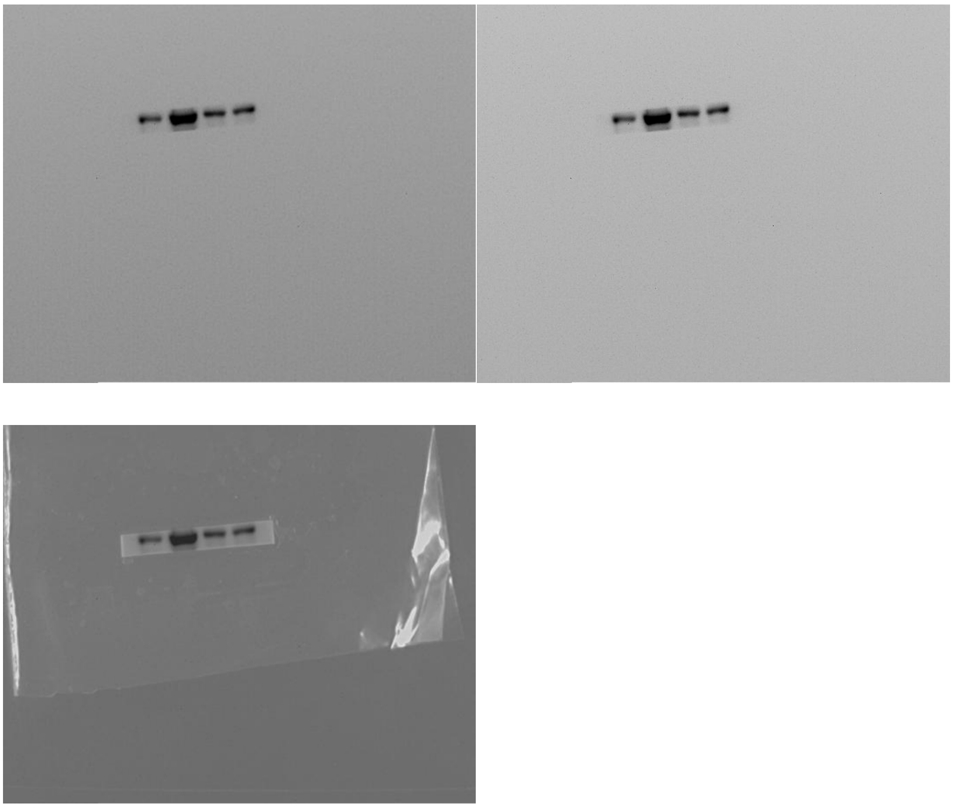

**β-actin (HepG2)**

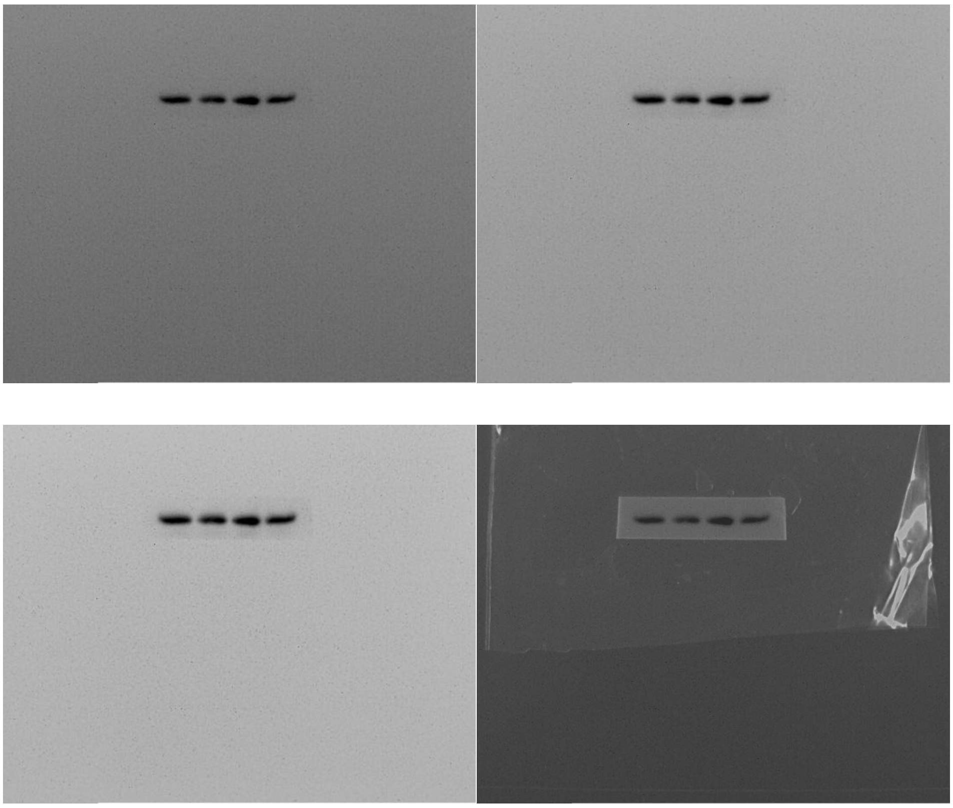

**MMP-2 (SK-Hep1)**

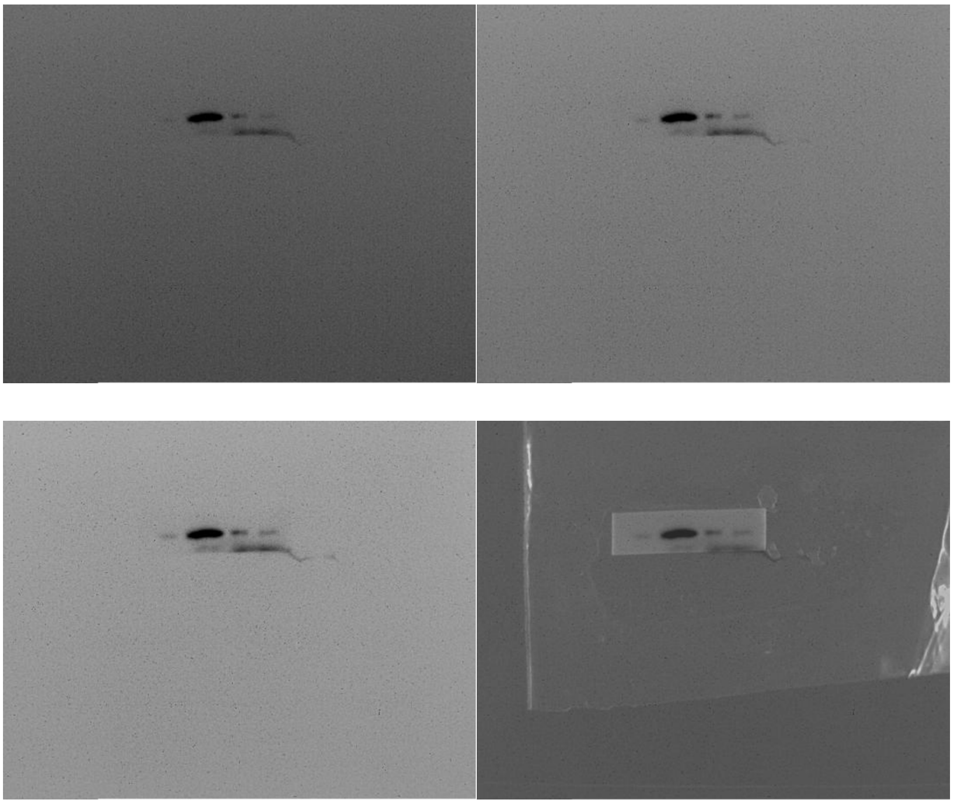

**β-actin (SK-Hep1)**

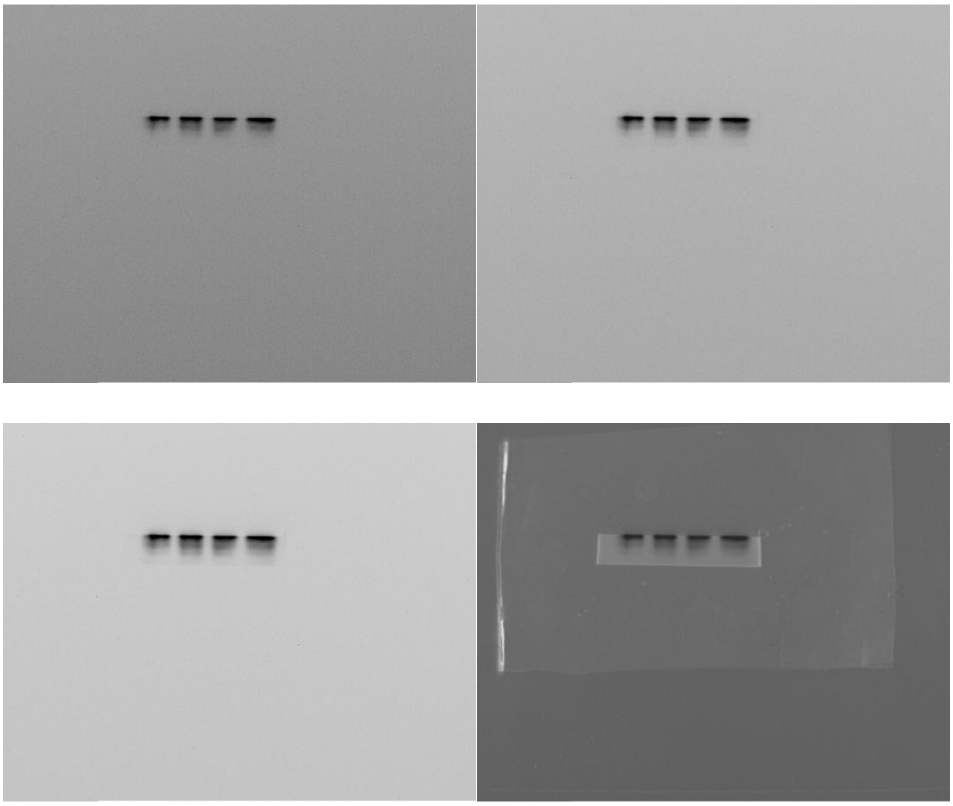

**Fig 8B1**

**MMP-9 (HepG2)**

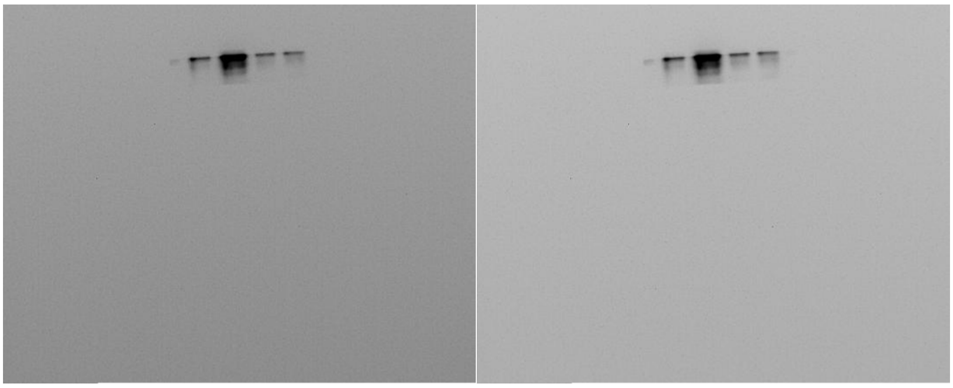

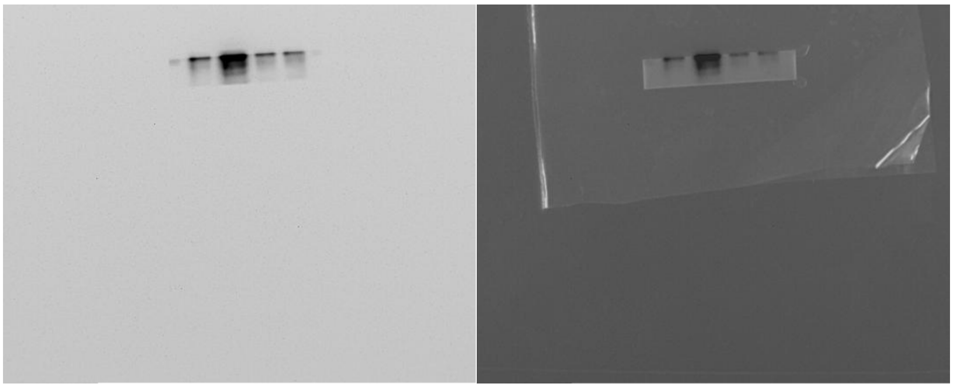

**β-actin (HepG2)**

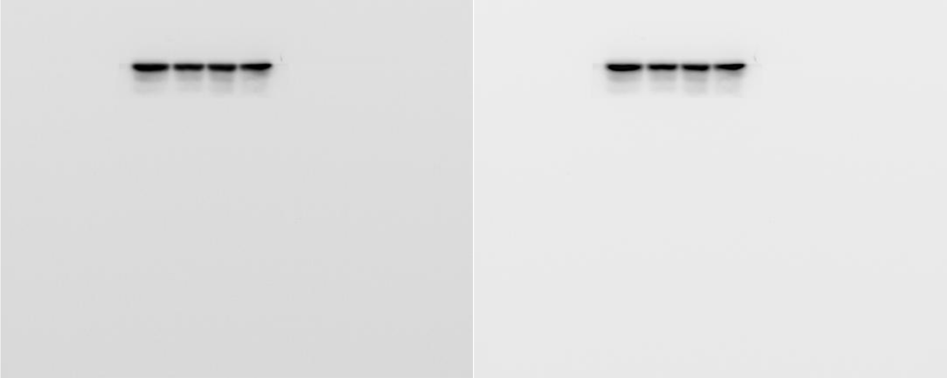

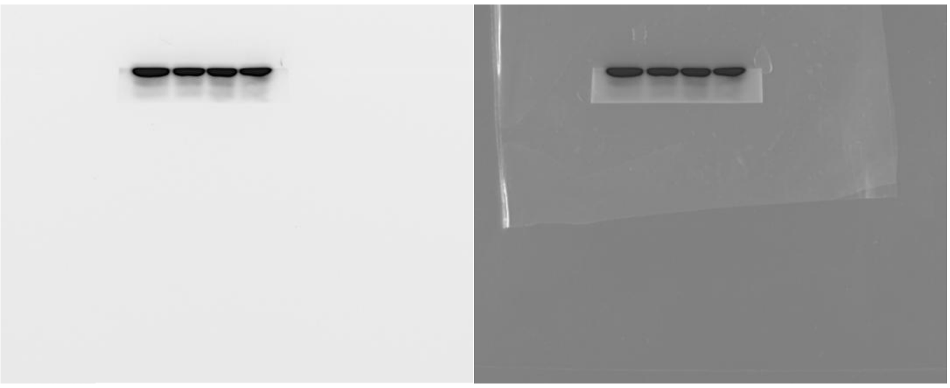

**MMP-9 (SK-Hep1)**

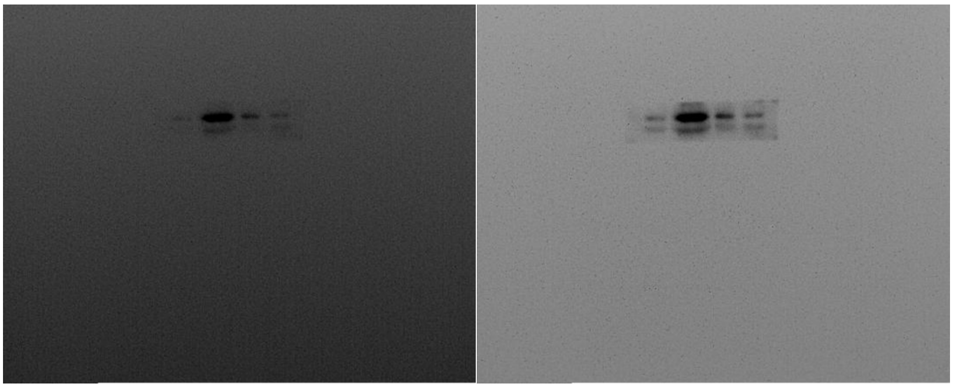

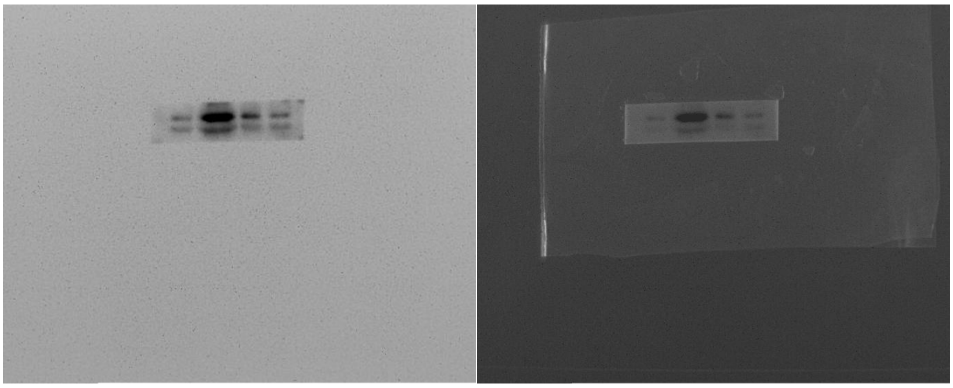

**β-actin (SK-Hep1)**

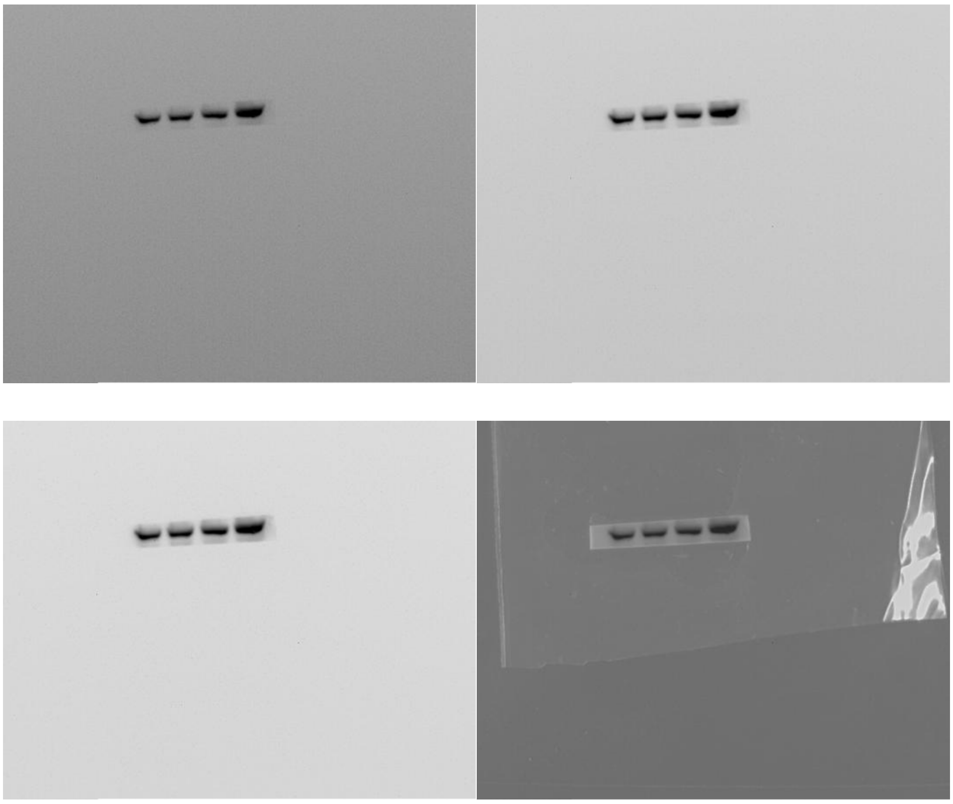

**Fig 8C1**

**p-Akt(Ser473) (HepG2)**

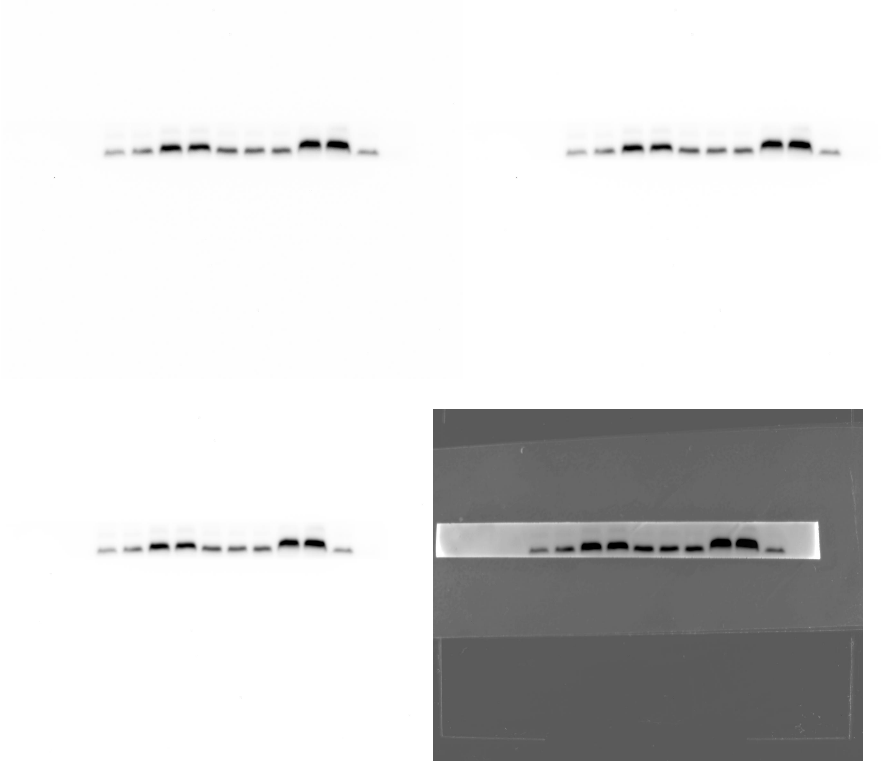

**t-Akt (HepG2)**

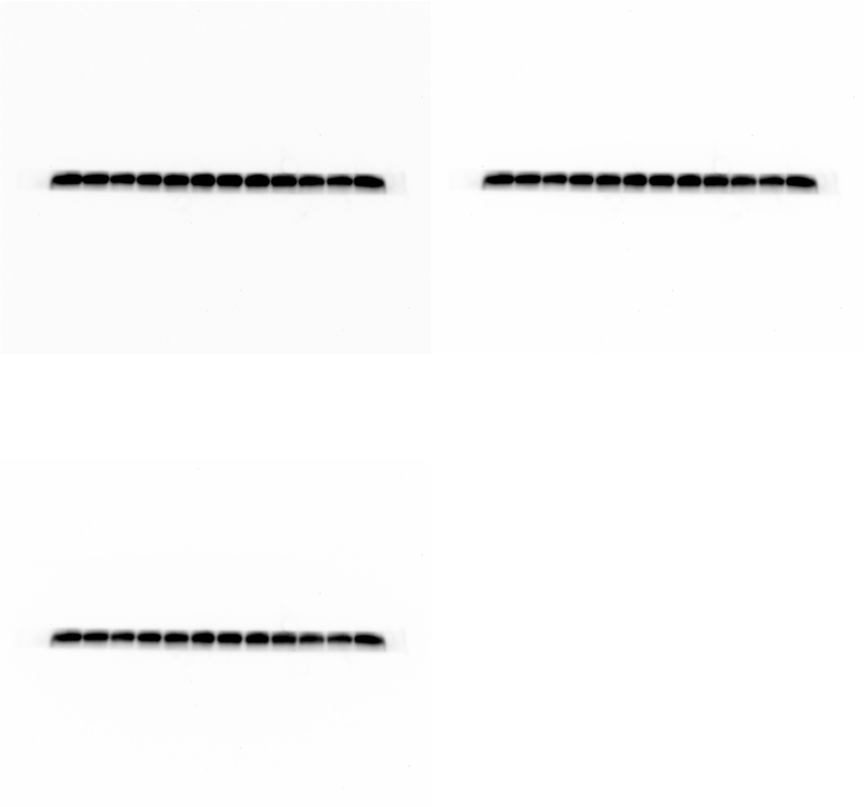

**β-actin (HepG2)**

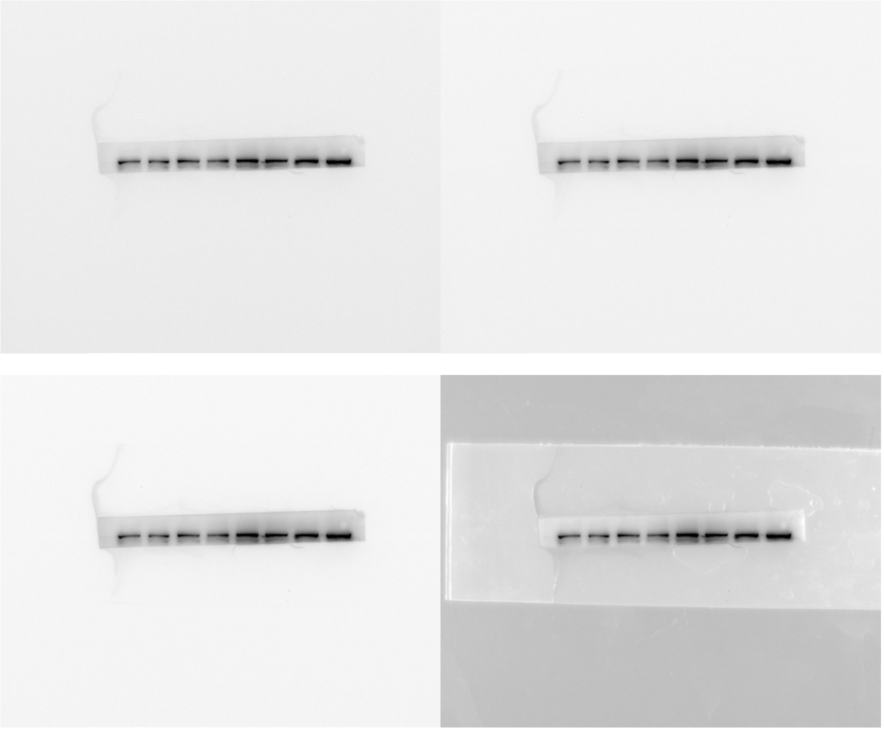

**p-Akt(Ser473) (SK-Hep1)**

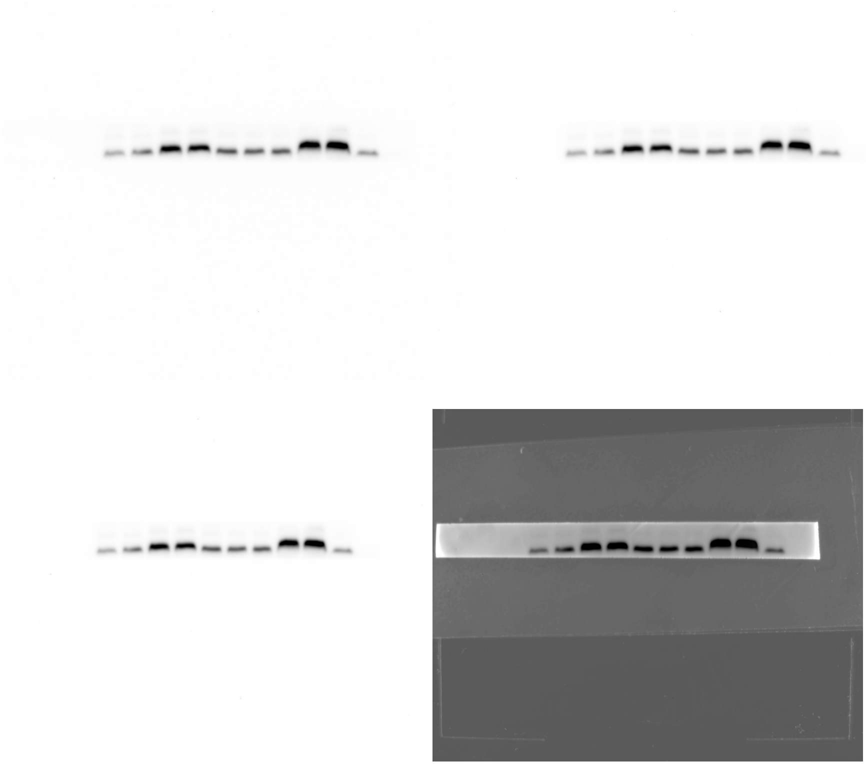

**t-Akt (SK-Hep1)**

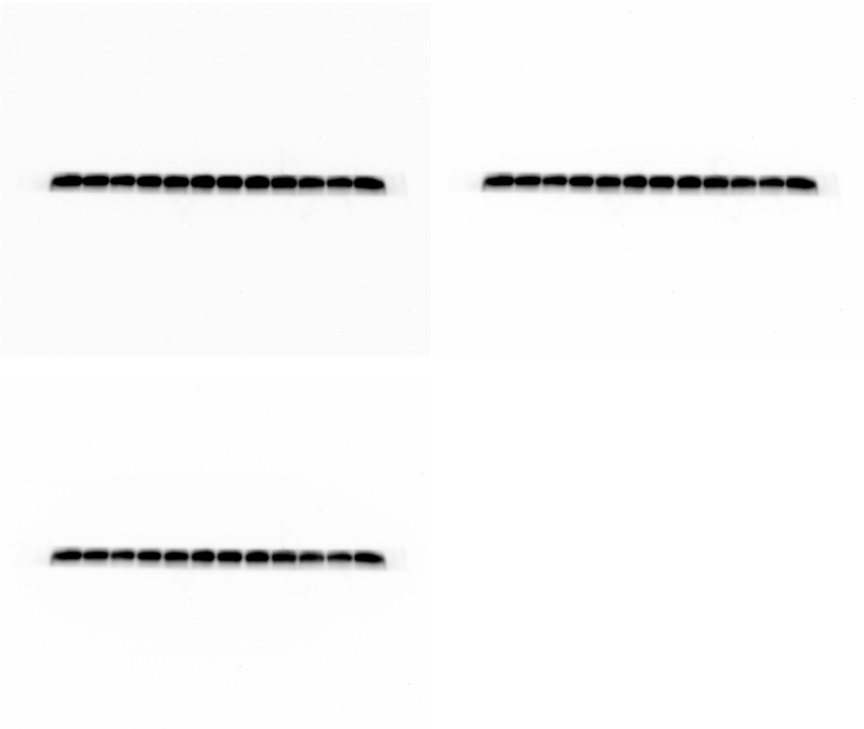

**β-actin (SK-Hep1)**

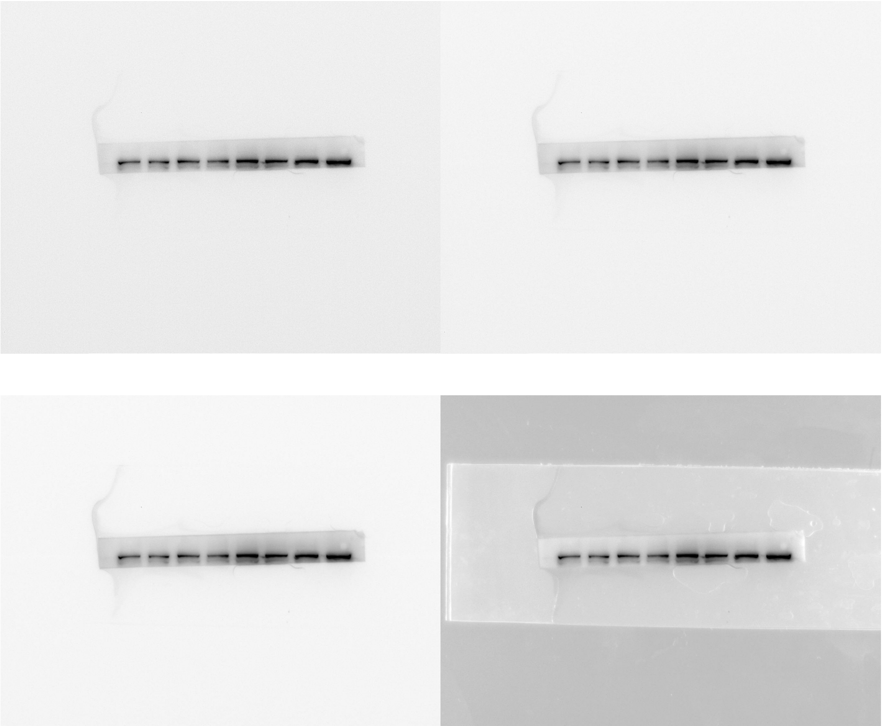

Remarks: Due to insufficient experimental funds, in the western blot assay, we cut the PVDF membrane into a membrane small enough to incubate the antibody according to the molecular weight of the incubated antibody and the protein molecular weight marker. In addition, protein samples of related research topics that were conducted simultaneously were added to the same gel for protein electrophoresis. We have made the above remarks in **Materials and methods** section of the manuscript.

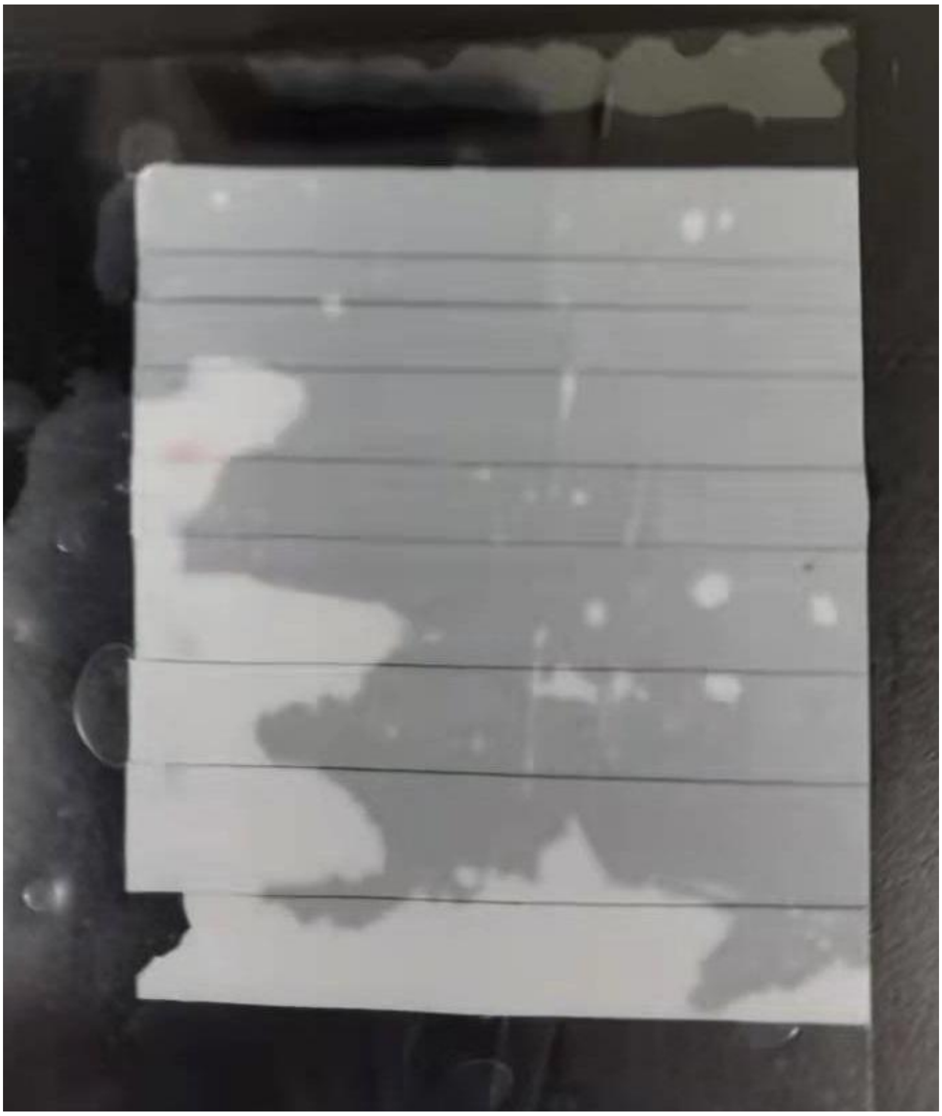

Supplement: Supplementary file 1 — Additional file 1. [file 12885_2022_9874_MOESM1_ESM.pdf]
